# Screening and Stability Validation of RT-qPCR Reference Genes in *Portulaca oleracea* L. in Diverse Tissues and Under Abiotic Stress Conditions

**DOI:** 10.3390/ijms27052276

**Published:** 2026-02-28

**Authors:** Jiahui Fang, Chenxin Fan, Jieshan Wang, Ming Yi, Ping Li, Mengyun Xu, Jian Yan

**Affiliations:** Key Laboratory of Agro-Environment in the Tropics, Ministry of Agriculture and Rural Affairs, Guangdong Provincial Key Laboratory of Eco-Circular Agriculture, Guangdong Engineering Research Centre for Modern Eco Agriculture, College of Natural Resources and Environment, South China Agricultural University, Guangzhou 510642, China; fangjiahui1109@gmail.com (J.F.); fanfan8752@163.com (C.F.); vvnn0305@163.com (J.W.); ymxjy20210116@163.com (M.Y.); liping2016@scau.edu.cn (P.L.)

**Keywords:** *Portulaca oleracea* L., reference genes, RT-qPCR, abiotic stress, gene expression normalization

## Abstract

Purslane (*Portulaca oleracea* L.) is an important plant species that has been increasingly used in functional gene studies and molecular analyses. However, reference genes that exhibit stable expression across multiple tissues and stress conditions have not been systematically validated in purslane, which limits the accuracy of reverse transcription quantitative PCR (RT-qPCR) based gene expression analyses. In this study, ten candidate reference genes from six gene families (*Actin*, *PP2A*, *CYP*, *eIF4A*, *Ubiquitin*, and *eIF5A*) were selected based on transcriptome data. A combination of bioinformatic analyses and experimental validation was employed to comprehensively characterize these candidates, including their physicochemical properties, chromosomal localization, phylogenetic relationships, gene structures, and promoter cis-acting elements. Furthermore, the expression stability of the candidate genes was systematically evaluated across different tissues (seed, root, stem, leaf, and flower) and under multiple stress treatments, including salinity, temperature stress, drought, and hormone treatments. Based on conventional PCR amplification specificity, melting curve analysis, Ct value distribution, and amplification efficiency, *ACT-2* and *eIF5A-1* were identified as the most stably expressed reference genes under diverse experimental conditions. This study provides reliable reference gene candidates for accurate normalization of gene expression in purslane and establishes a systematic framework for reference gene selection in non-model plant species.

## 1. Introduction

Purslane (*Portulaca oleracea* L.), a succulent annual herb belonging to the Portulacaceae family, is widely distributed across the world [1]. Its prolific reproductive potential and exceptional adaptability have contributed to its recognition as the eighth most frequent plant [2]. Acknowledged for its dual nutritional and pharmacological value, purslane has been listed by the World Health Organization among the most widely used medicinal plants worldwide [3,4]. Its diverse pharmacological activities are primarily attributed to a wide range of secondary metabolites, including polysaccharides, flavonoids, organic acids, terpenoids, monoterpene glycosides, and alkaloids, as well as its high levels of vitamin C, β-carotene, and α-linolenic acid compounds that confer significantly higher nutritional value compared to most wide consumed vegetables [5,6,7]. With the completion of a high-quality, chromosome-level genome assembly, it was reported that *Portulaca oleracea* L. is a diploid species possessing 52 chromosomes (2n = 52), and increasing attention has been devoted to elucidating the gene functions underlying its genetic resources for functional and applied research [8]. However, accurate RT-qPCR-based gene expression analysis in purslane remains constrained by the absence of systematically validated reference genes. As a consequence of this growing research interest, accurate and reproducible gene expression analysis has become increasingly important for functional studies in purslane. In functional gene validation and expression dynamics studies, reverse transcription quantitative PCR (RT-qPCR) has been widely employed due to its high sensitivity and specificity [9]. However, the accuracy of RT-qPCR data is highly dependent on the expression stability of the reference genes used for normalization; improper selection of reference genes can lead to biased results or even misinterpretation [10,11,12,13]. Therefore, systematically identifying reference genes that exhibit high expression stability across various tissues and under multiple stress conditions is essential, as it provides a reliable normalization standard for accurate gene quantification and subsequent functional studies.

RT-qPCR offers high sensitivity and a wide dynamic range; its accuracy and biological relevance can only be ensured when rigorous normalization strategies, such as the use of stably expressed reference genes, are employed to control for extraction and handling errors [14]. An ideal reference gene should exhibit stable expression across different tissue types, developmental stages, and experimental conditions, remaining unaffected by external environmental changes [15]. Traditionally, commonly used reference genes include *Actin*, *Tubulin*, *Ubiquitin*, *GAPDH* (*glyceraldehyde-3-phosphate dehydrogenase*), and *EF1α* (*elongation factor 1-alpha*) [16,17,18,19,20,21]. Before the widespread application of genomic technologies, these genes were commonly selected as reference genes due to their central roles in maintaining essential cellular processes such as structural integrity and primary metabolism. As a result, they were often referred to as “housekeeping genes” or “reference genes”.

However, increasing evidence indicates that the expression stability of these traditional housekeeping genes is not universal but highly dependent on species, tissue types, developmental stages, and experimental conditions. Even among phylogenetically related species, reference gene performance can differ substantially. Species-specific differences in reference gene suitability have been reported even among closely related plant species. For instance, systematic RT-qPCR evaluation in tomato (*Solanum lycopersicum*) under fruit ripening and mechanical wounding conditions identified *GAPDH* as the most stable reference genes [22]. In contrast, systematic validation studies on the closely related potato (*Solanum tuberosum*) demonstrated that *GAPDH* exhibited substantial expression variability [23]. Moreover, differences in experimental context and validation strategy can also lead to the identification of distinct optimal reference genes, even within the same species. For example, in tomato, reference gene evaluation conducted under fruit-specific and wounding-related experimental conditions favored *GAPDH* and *EF1α* [22], whereas RT-qPCR-based validation focusing on developmental processes identified non-classical reference genes as more suitable internal controls, while several commonly used housekeeping genes performed poorly [24]. These observations collectively highlight that reference gene suitability is strongly dependent on tissue type, developmental stage, and experimental conditions, rather than species identity alone.

In light of these findings, species-specific validation becomes particularly important for plant species that are increasingly used in functional and stress-related studies but lack established reference genes. To date, no systematic validation of reference genes has been reported for purslane. By identifying and validating stable internal controls for purslane, our work addresses the lack of systematically validated reference genes and provides a robust basis for reliable normalization in gene expression analyses in purslane as an emerging model and crop species.

In this study, we aimed to systematically identify and validate suitable reference genes for RT-qPCR normalization in purslane across multiple tissues and stress conditions. Based on transcriptome data, genes with relatively stable expression across different tissues were first identified, and their gene family affiliations were subsequently assigned according to functional annotation. These candidate genes were then subjected to comprehensive bioinformatic analyses, including characterization of physicochemical properties, chromosomal distribution, phylogenetic relationships, gene structures, and promoter cis-acting elements, followed by experimental assessment of expression stability across different tissues and under diverse abiotic and hormone-related stress treatments. Through this integrated approach, stable reference genes were identified to provide a reliable normalization framework for accurate gene expression analysis in purslane.

## 2. Results

### 2.1. Identification and Physicochemical Characterization of Candidate Reference Genes in Purslane

Based on transcriptome data from different tissues, ten candidate reference genes showing relatively consistent expression patterns across root, stem, leaf, and flower tissues were selected for further investigation. The physicochemical properties of the proteins encoded by these candidate genes were subsequently analyzed (Table 1). To facilitate interpretation, gene names and concise functional annotations were provided for each Gene ID in Table 1, and closely related family members were distinguished using numerical suffixes. The predicted protein lengths ranged from 139 to 3750 amino acids (aa), with *ACT-2* encoding the shortest protein (139 aa) and *Ubi-1* encoding the longest protein (3750 aa). The theoretical molecular weights of the candidate proteins varied from 16.2 kDa (*ACT-2*) to 407.78 kDa (*Ubi-1*). Theoretical isoelectric point (pI) values ranged from 4.97 to 11.72. Instability index predictions showed that three candidate proteins had values below 40, whereas others exhibited higher instability indices. In addition, the aliphatic index varied among the candidate proteins, and all candidates displayed negative GRAVY values, indicating that they are predominantly hydrophilic and likely soluble proteins.

### 2.2. Chromosomal Distribution, Gene Structure, and Conserved Domain Analysis

**Figure 1:**
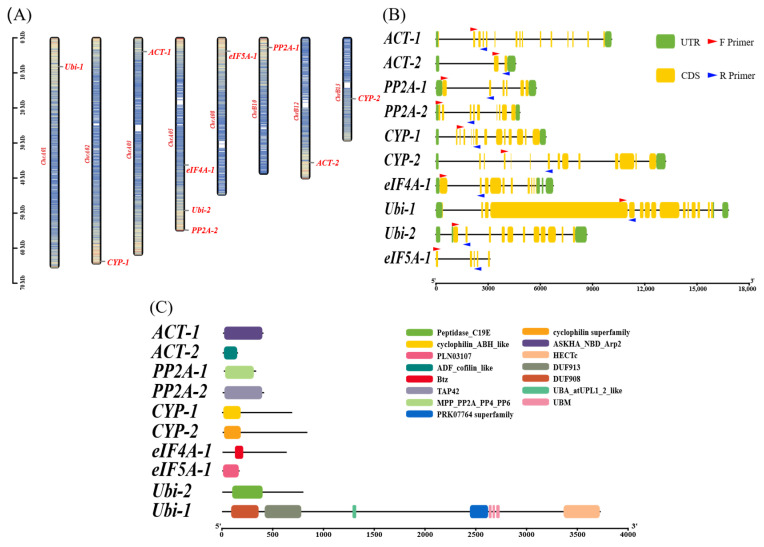
Genomic characterization of the ten candidate reference genes in purslane. (**A**) Chromosomal localization of the candidate reference genes. Chromosome numbers are shown on the left side, and the gene names are shown on the right side; (**B**) Gene structures of the candidate reference genes. Green boxes represent untranslated regions (UTRs), yellow boxes indicate exons, and black lines denote introns. Red and blue arrows indicate the positions of forward and reverse primers, respectively; (**C**) Conserved domain architectures of the candidate reference genes. Different domains are represented by colored boxes.

Based on the chromosomal location information of the ten candidate reference genes in purslane, a chromosomal distribution map was generated using TBtools (v2.376) [25,26] (Figure 1A). The results showed that the ten candidate reference genes were distributed across eight chromosomes in the purslane genome. Among them, three candidate reference genes were located on chromosome 5, whereas chromosomes 1, 2, 3, 8, 10, 12, and 13 each contained one candidate reference gene. The exon–intron structures of the candidate reference genes were further analyzed (Figure 1B). The results revealed considerable variation in gene structure among the candidates. Four candidate reference genes contained fewer than ten exons, while the remaining six genes harbored more than ten exons. Notably, *Ubi-1* possessed the highest number of exons (16) and introns (15). Conserved domain analysis revealed that all candidate reference genes contained the characteristic domains corresponding to their annotated gene families (Figure 1C). For example, *ACT-2* harbored an ADF/cofilin-like domain, which is commonly associated with *Actin* proteins, whereas *eIF5A-1* contained the conserved *eIF5A* domain involved in translational regulation. These results indicate that the predicted proteins possess intact and family-consistent domain architectures.

### 2.3. Expression Characteristics and Preliminary Screening of Candidate Reference Genes

**Figure 2:**
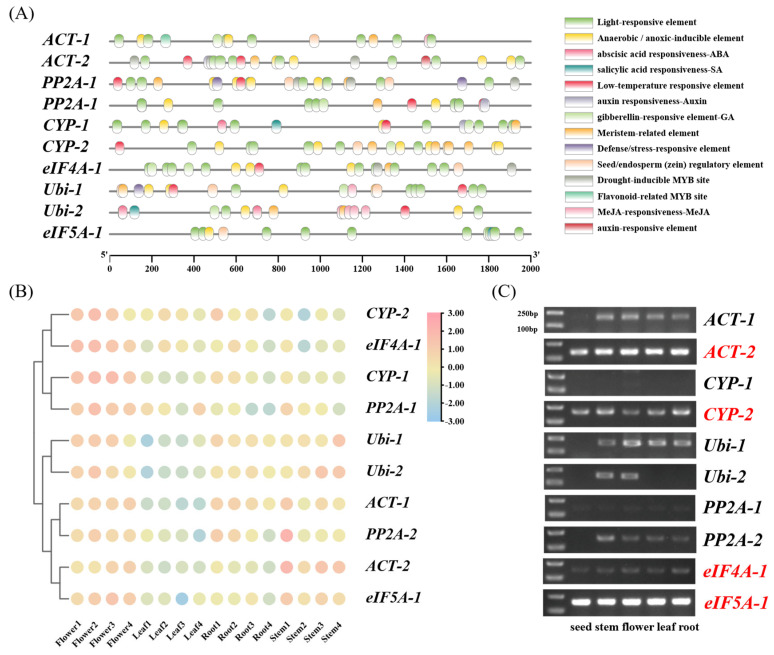
Expression characteristics and preliminary screening of candidate reference genes in purslane. (**A**) Cis-acting elements regulatory element analysis of the promoter regions of candidate reference genes in purslane; (**B**) Gene expression levels were obtained from transcriptome data and represented as TPM values. The displayed genes were selected based on relatively small expression differences among tissues. Heatmaps were generated based on TPM values to visualize expression patterns across different tissues. Color intensity represents relative expression levels, and genes were clustered using hierarchical clustering; (**C**) Expression of candidate reference genes in five tissues (seed, root, stem, leaf, and flower) examined by conventional PCR and visualized by 1% (*w*/*v*) agarose gel electrophoresis.

To characterize the regulatory features of candidate reference genes, cis-acting elements in the promoter regions were analyzed using the PlantCARE database (Figure 2A). A total of 16 stress-responsive cis-acting elements were identified in the promoters of the ten candidate reference genes. These elements could be broadly classified into hormone-responsive elements (including abscisic acid, gibberellin, auxin, and methyl jasmonate), abiotic stress responsive elements (such as drought, low-temperature, and anaerobic response elements), and growth and development-related elements (including light-responsive, meristem-related, and seed/endosperm-associated elements).

Based on transcriptome data, heatmap visualization was performed to examine the expression patterns of the selected candidate reference genes in purslane. The results showed that these genes exhibited relatively small differences in transcript abundance across the analyzed tissues, without obvious tissue-specific expression patterns (Figure 2B). To assess expression patterns and amplification specificity of candidate reference gene primers in different tissues, conventional PCR was performed using cDNA from five key tissues, including seeds, roots, stems, leaves, and flowers. The amplified products were examined by agarose gel electrophoresis to verify product size and specificity (Figure 2C). Full-length, uncropped agarose gel electrophoresis images corresponding to Figure 2C are provided in the Supplementary Information (Figure S1). The results showed that *CYP-1* and *PP2A-1* exhibited very low expression levels, with barely detectable bands across all tissues. Although *PP2A-2*, *Ubi-1*, *Ubi-2*, and *ACT-1* showed detectable expression, their amplification was limited to a subset of tissues and therefore these genes were excluded from further analysis. In contrast, *ACT-2*, *CYP-2*, *eIF4A-1*, and *eIF5A-1* were consistently amplified across all examined tissues. Notably, *eIF5A-1* and *ACT-2* produced the most prominent bands, indicating higher detection sensitivity. Based on this preliminary evaluation, four genes that produced clear and reproducible PCR amplification across different tissues were retained for subsequent analyses.

### 2.4. RT-qPCR Analysis of Candidate Reference Gene Expression

**Figure 3:**
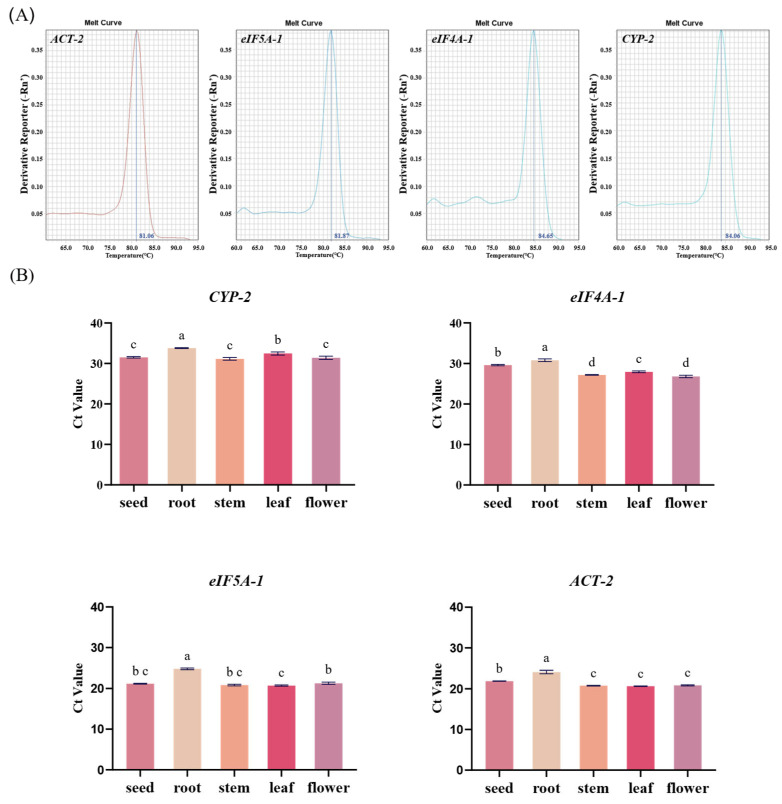
Melting curve analysis and Ct value distribution of candidate reference genes in purslane. (**A**) Melting curves of four candidate reference genes obtained from leaf tissue, showing single sharp peaks and indicating specific amplification; (**B**) Ct value distributions of four candidate reference genes across five tissues (seed, root, stem, leaf, and flower). Ct values reflect relative expression levels of the candidate genes. Data are presented as mean ± standard deviation (n = 3). Statistical analysis was performed using one-way ANOVA. Different letters above the bars indicate significant differences (*p* < 0.05).

To further evaluate the amplification specificity and detection performance of candidate reference gene primers, RT-qPCR was conducted to examine their expression characteristics in different purslane tissues. Amplification specificity was assessed by melting curve analysis of the RT-qPCR products. As shown in Figure 3A, representative melting curves obtained from leaf tissue samples exhibited a single sharp peak for all reactions, indicating specific amplification of a single product without non-specific amplification or primer-dimer formation. After confirming primer specificity, the expression levels of candidate reference genes in five tissues (seed, root, stem, leaf, and flower) were compared based on cycle threshold (Ct) values. Figure 3B shows the Ct value distributions of four candidate reference genes obtained from three technical replicates. All four genes were successfully amplified across the five tissues examined. Among them, *ACT-2* and *eIF5A-1* exhibited overall lower Ct values, whereas *CYP-2* and *eIF4A-1* showed relatively higher Ct values, indicating differences in detection sensitivity among the candidate genes. Based on these RT-qPCR expression characteristics, *ACT-2* and *eIF5A-1* were retained for subsequent analyses.

### 2.5. Evaluation of Reference Gene Expression Stability Under Various Stress Conditions

**Figure 4:**
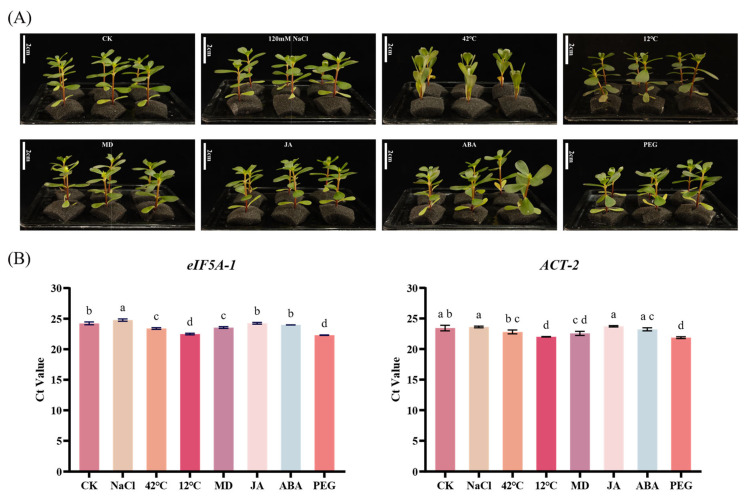
Expression characteristics of candidate reference genes under abiotic stress in purslane. (**A**) Experimental design of seven abiotic stress treatments, including 120 mM NaCl, heat stress at 42 °C, cold stress at 12 °C, mechanical damage (MD), 8% PEG6000, and hormone treatments with 1 μM jasmonic acid (JA) and 1 μM abscisic acid (ABA), along with an untreated control (CK); (**B**) Ct value distributions of *eIF5A-1* and *ACT-2* under different stress treatments. Ct values reflect relative expression levels of the candidate reference genes. Data are presented as mean ± standard deviation (n = 3). Statistical analysis was performed using one-way ANOVA. Different letters above the bars indicate significant differences (*p* < 0.05).

To assess the expression stability of the candidate reference genes, seven abiotic stress treatments were applied: 120 mM NaCl, heat stress at 42 °C, cold stress at 12 °C, MD, 8% PEG6000, and hormone treatments with 1 μM JA and 1 μM ABA, with an untreated control (CK) included (Figure 4A). After 24 h of treatment, RT-qPCR data were collected and analyzed (Figure 4B). The Ct values for *eIF5A-1* ranged from 22.30 ± 0.04 (PEG treatment) to 24.77 ± 0.19 (NaCl treatment), while those for *ACT-2* ranged from 21.87 ± 0.15 (PEG treatment) to 23.72 ± 0.10 (JA treatment). Under all seven stress conditions, both genes showed minimal expression variation, with no significant differences compared to the control (CK), demonstrating their consistent expression stability and thus validating their suitability as stable indicators for accurate cDNA quantification.

### 2.6. Dilution Curve Analysis of Candidate Reference Gene Amplification Performance

**Figure 5:**
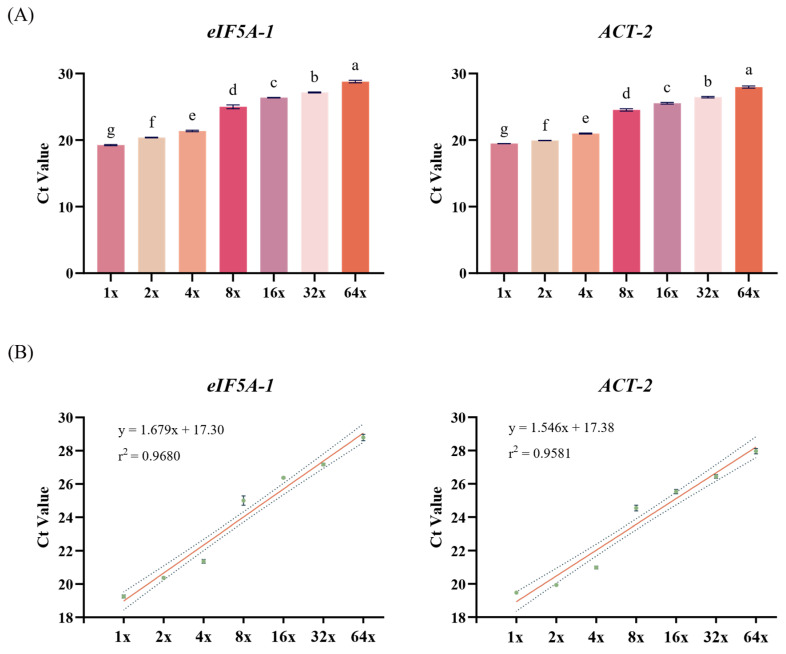
Dilution curve analysis of candidate reference gene amplification performance in purslane. (**A**) Ct value distributions of *eIF5A-1* and *ACT-2* across a serial dilution series (1×, 2× 4×, 8×, 16×, 32×, and 64×). Data are presented as mean ± standard deviation (n = 3). Statistical analysis was performed using one-way ANOVA. Different letters above the bars indicate significant differences (*p* < 0.05). (**B**) Linear regression plots of Ct values against dilution factors. Regression equations and coefficients of determination (R^2^) are indicated on the graph.

To further assess the amplification performance of the two retained candidate reference genes, *eIF5A-1* and *ACT-2*, a serial dilution experiment was performed. cDNA from the same sample was serially diluted by factors of 1×, 2×, 4×, 8×, 16×, 32×, and 64×, followed by RT-qPCR amplification at each dilution point, and the corresponding Ct values were recorded (Figure 5A). The results showed a clear linear increase in Ct values with increasing dilution factors for both genes. Linear regression analysis was performed by plotting Ct values against the serial dilution points of a two-fold dilution series (1×–64×) to assess amplification linearity to calculate the coefficient of determination (R^2^) (Figure 5B). The R^2^ values for *eIF5A-1* and *ACT-2* were 0.9680 and 0.9581, respectively, both exceeding 0.95, indicating strong linearity between Ct values and template dilution. These results demonstrate that the RT-qPCR assays for *eIF5A-1* and *ACT-2* exhibit reliable amplification linearity across a wide range of template concentrations. Together, these results indicate that the RT-qPCR assays for *eIF5A-1* and *ACT-2* respond consistently to changes in template concentration, supporting their technical suitability for use as reference genes.

### 2.7. Phylogenetic Relationships and Prediction of Protein 3D Structure

**Figure 6:**
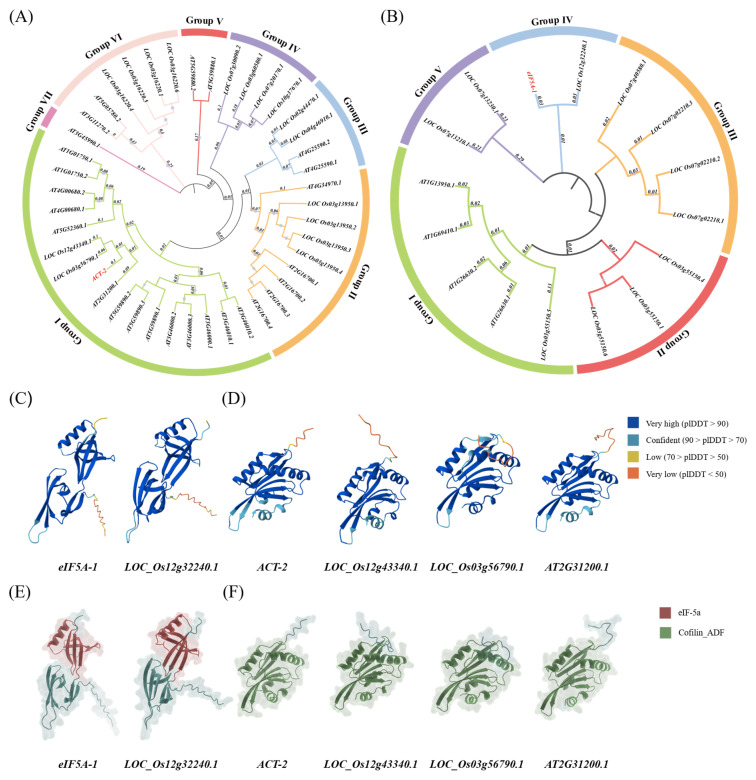
Phylogenetic relationships of *ACT-2* and *eIF5A-1* and predicted protein structural features. (**A**,**B**) Phylogenetic trees of *ACT-2* (**A**) and *eIF5A-1* (**B**) constructed using the Neighbor-Joining (NJ) method in MEGA 12 (64-bit, Windows). Bootstrap analysis was performed with 1000 replicates. Different colors indicate distinct phylogenetic groups, and *ACT-2* and *eIF5A-1* are marked with red text; (**C**,**D**) AlphaFold-predicted three-dimensional structures of *ACT-2*, *eIF5A-1*, and their homologous proteins, colored according to predicted local distance difference test (pLDDT) scores to indicate model confidence; (**E**,**F**) Conserved domain architectures of the corresponding proteins, illustrating the relative positions and extents of annotated conserved domains along the protein sequences.

To examine the phylogenetic relationships of the two retained candidate reference genes across representative plant species, their encoded protein sequences were used as queries to identify homologous proteins in Arabidopsis (*Arabidopsis thaliana* L.) and rice (*Oryza sativa* L.), and phylogenetic trees were constructed. In the phylogenetic tree of *ACT-2*, a total of 16 homologs from rice and 26 from Arabidopsis were included (Figure 6A), whereas the phylogenetic tree of *eIF5A-1* contained 11 rice and 4 Arabidopsis homologs (Figure 6B). These homologs were defined based on both sequence similarity and conserved domain annotation to ensure reliable phylogenetic inference. Based on the phylogenetic topology, *ACT-2* homologs could be divided into seven distinct clades, whereas *eIF5A-1* homologs formed five distinct clades. *ACT-2* clustered with its homologs from Arabidopsis (*AT2G31200.1*) and rice (*LOC_Os03g56790.1* and *LOC_Os12g43340.1*) within the same clade (Group I), indicating a conserved evolutionary relationship across representative dicot and monocot species. In contrast, *eIF5A-1* clustered with its closest homolog from rice (*LOC_Os12g32240.1*) in an independent clade (Group IV), suggesting a distinct but conserved evolutionary lineage.

At the protein structural level, 3D structures were predicted for *ACT-2*, *eIF5A-1*, and their closely related homologous proteins using AlphaFold [27]. The predicted structures of proteins are shown in Figure 6C, with residues colored according to their predicted local distance difference test (pLDDT) scores, reflecting the confidence of the structural models. The high-confidence regions were predominantly distributed within the conserved core regions of each protein. To further characterize the structural organization, the positions of conserved domains within the protein sequences were visualized schematically (Figure 6D). Comparison of conserved domain architectures revealed clear differences between the two candidate reference genes. In *eIF5A-1* and its homologous proteins, Eukaryotic elongation factor 5A hypusine, DNA-binding OB fold (PF01287) conserved domain occupied approximately half of the protein length. In contrast, *ACT-2* and its homologs were characterized by a Cofilin/tropomyosin-type actin-binding protein (PF00241) conserved domain that spanned the majority of the protein sequence, indicating a more compact domain organization. Notably, the relative positions and coverage of these conserved domains were highly consistent among homologous proteins within each group. The domain architecture analysis showed that the candidate reference genes and their homologs shared similar conserved domain compositions and domain positions along the protein sequences.

## 3. Discussion

*Portulaca oleracea* L. is not only a nutrient-rich vegetable commonly consumed in daily diets, but also a plant rich in diverse bioactive compounds and with strong tolerance to adverse environmental conditions, which has attracted increasing attention in medicine, ecological restoration, and basic research [28,29,30]. In recent years, the availability of a high-quality genome sequence and assembly has provided a comprehensive genetic framework for purslane, revealing its genetic resources and evolutionary origins and laying an important foundation for molecular breeding and functional studies [8]. Meanwhile, transcriptome analyses have generated a dynamic “expression atlas”, systematically illustrating gene expression patterns across different tissues, developmental stages, and stress conditions. When integrated with metabolomic data, these resources have further facilitated the investigation of biosynthetic pathways of key secondary metabolites [8,31,32,33].

Although multi-omics approaches provide abundant resources for the identification of candidate functional genes, translating these datasets into reproducible and comparable quantitative results still relies on accurate gene expression analyses. Reverse transcription quantitative PCR (RT-qPCR) is widely used due to its high sensitivity and specificity; however, to minimize technical and biological variation, reliable normalization using reference genes with relatively consistent expression patterns across experimental conditions is essential [34,35,36]. Previous studies have shown that commonly used reference genes often display substantial expression variability among different plant species, tissues, and environmental treatments [37,38,39]. Therefore, the present study aimed to systematically evaluate and identify candidate reference genes exhibiting relatively consistent expression characteristics across multiple tissues and abiotic stress conditions in purslane, thereby providing a reliable reference for gene expression analyses in this species.

Based on comparative transcriptome analyses, a total of ten candidate reference genes exhibiting relatively stable expression across different tissues were preliminarily identified in Portulaca oleracea. These genes belong to six distinct gene families, including *Actin* [40,41], *PP2A* [42], *CYP* [43], *eIF4A* [44,45], *Ubiquitin* [46], and *eIF5A* [47]. Previous studies have demonstrated that members of these gene families are widely employed as reference genes in various plant species and generally display favorable expression stability under diverse experimental conditions, indicating their potential suitability as internal controls [48,49,50,51,52,53]. Accordingly, one or two representative genes with relatively high expression stability were selected from each family for further analysis. At the genomic level, chromosomal localization analysis revealed that these candidate reference genes are distributed across different chromosomes in the *P. oleracea* genome and do not form obvious gene clusters (Figure 1A), suggesting that they are unlikely to have arisen from recent tandem duplication events. This dispersed genomic distribution may reduce the potential impact of copy number expansion on expression variability. On this basis, gene structure (Figure 1B) and physicochemical properties of the encoded proteins (Table 1) were further analyzed to characterize these candidate reference genes at the molecular level. Notable differences in gene structure were observed among the candidates: three genes contained relatively few exons, whereas the remaining genes harbored a larger number of exons, indicative of more complex gene architectures. In addition, variations in several physicochemical properties were detected among the encoded proteins.

Domain analysis showed that all candidate reference genes possess distinct conserved domains, most of which are located near the N-terminal region (Figure 1C). Promoter cis-acting elements analysis further revealed that all ten candidate reference genes contain both light-responsive elements and anaerobic/anoxic-inducible elements. Light-responsive cis-acting elements are widely distributed in plant promoters; representative motifs such as the G-box, characterized by an ACGT core sequence, can be recognized by key light-signaling transcription factors including HY5 and PIFs, thereby mediating light-dependent transcriptional regulation and frequently appearing in promoter annotations of diverse plant genes [54,55]. In addition, plants frequently experience low-oxygen conditions under circumstances such as waterlogging, rhizosphere hypoxia, and limited oxygen diffusion within tissues, making anaerobic/anoxic-responsive cis-acting elements a common feature of plant promoters. Classical studies using anaerobic metabolism-related genes, such as alcohol dehydrogenase (ADH), as model systems have elucidated the modular regulatory characteristics of anaerobic-responsive cis-acting elements [56]. Collectively, these two classes of cis-acting elements are broadly distributed in plant promoters and function primarily in sensing fluctuations in environmental and physiological conditions rather than acting as strict transcriptional on–off switches. Previous studies have shown that even genes associated with fundamental cellular processes often harbor multiple stress- or environment-related cis-acting elements, reflecting the complexity and flexibility of transcriptional regulation in plants. Promoter cis-acting element analysis was included to provide regulatory context and to illustrate the complexity and flexibility of transcriptional regulation in plant genes under diverse environmental and physiological conditions.

In this study, heatmap analysis based on transcriptome data was employed to examine the expression patterns of the candidate reference genes (Figure 2B). The results showed that the transcript abundance of these genes was relatively comparable across different tissue samples, with no apparent tissue-specific expression trends. These findings provide transcriptome-level support for the preliminary selection of candidate reference genes, suggesting that they exhibit relatively consistent expression across different tissues. It should be noted, however, that transcriptome analysis primarily reflects relative gene expression levels under specific experimental conditions and sequencing platforms, and the results may be influenced by factors such as sample source, sequencing depth, and normalization strategies [57,58,59]. Therefore, to further enhance the reliability of reference gene selection, conventional PCR, RT-qPCR, and expression analyses under multiple experimental conditions were subsequently conducted for systematic validation. Through conventional PCR amplification followed by agarose gel electrophoresis, four candidate genes were retained for subsequent analyses because they produced clear, reproducible single amplification bands across all tested tissues (Figure 2C), indicating sufficient transcript abundance and reliable primer performance. In contrast, several other candidate genes exhibited faint or inconsistent bands in one or more tissues. This phenomenon is likely attributable to relatively low transcript abundance under the tested conditions or reduced amplification efficiency of the corresponding primer pairs. Importantly, although faint bands do not necessarily indicate intrinsic expression instability of a gene, low or barely detectable expression levels can compromise the robustness and reproducibility of RT-qPCR normalization, particularly when comparing multiple tissues or stress conditions. Therefore, genes showing weak or inconsistent amplification were excluded from further analysis, as they are less suitable for use as reference genes in quantitative expression studies requiring reliable detection across all samples. Among these four candidate genes, *ACT-2* and *eIF5A-1* displayed noticeably stronger and sharper bands compared with the other candidates, likely reflecting their relatively higher transcript abundance and favorable primer amplification performance. Consistent with these observations, RT-qPCR analysis revealed that *ACT-2* and *eIF5A-1* exhibited overall lower Ct values across five different tissues (seed, root, stem, leaf, and flower) together with relatively limited Ct variation among samples. Importantly, the combination of moderate transcript abundance and low expression variability across tissues supports their suitability as reference genes, as stable and reliably detectable expression is essential for accurate RT-qPCR normalization (Figure 3B).

In RT-qPCR analysis, the cycle threshold (Ct) value reflects the relative abundance of the target transcript and is widely used as a primary indicator for gene expression quantification. According to the MIQE guidelines, candidate reference genes should not be presumed to be stable a priori; instead, their expression stability must be experimentally validated under the specific tissues and treatment conditions being investigated [60]. Therefore, the limited Ct variation observed for these four candidate genes supports their suitability as stable reference genes under the tested conditions [14]. Moreover, given that environmental stresses can substantially influence plant transcriptional activity, previous studies have emphasized that reference genes selected solely under normal growth conditions may be insufficient for complex experimental designs. Systematic evaluation of candidate reference genes under different stress treatments is thus essential to improve the accuracy and comparability of RT-qPCR data normalization [19,20]. Accordingly, the present study further assessed the expression performance of candidate reference genes under multiple abiotic stress conditions. Based on RT-qPCR analysis across different tissues, *ACT-2* and *eIF5A-1* exhibited relatively low Ct values with minimal variation and were therefore selected as reference genes for subsequent experiments. In this study, a total of seven stress treatments were applied (Figure 4A), which can be broadly classified into three categories: abiotic stress, hormone treatment, and physical stress. These treatments included 120 mM NaCl, high temperature (42 °C), low temperature (12 °C), mechanical damage (MD), 8% PEG6000, 1 μM jasmonic acid (JA), and 1 μM abscisic acid (ABA). Phenotypic observations confirmed that only high-temperature stress induced pronounced physiological responses in purslane seedlings. Nevertheless, the Ct values of the selected reference genes did not exhibit evident treatment-specific shifts, indicating that their expression levels remained relatively stable across the tested stress conditions.

Based on the above analyses, *ACT-2* and *eIF5A-1* were identified as candidate reference genes with strong application potential for RT-qPCR analyses in purslane. *ACT-2* belongs to the *Actin* gene family, whereas *eIF5A-1* is a member of the *eIF5A* gene family. *Actin* encodes actin proteins, which are core components of the cytoskeleton and play fundamental roles in maintaining cell structure and facilitating intracellular transport. Consistent with their roles as core cytoskeletal components, *Actin* genes have frequently been reported to exhibit relatively consistent expression patterns across diverse plant species [61]. For instance, *Actin* displayed low expression variability under multiple abiotic stress and hormone treatments in celery (*Apium graveolens* L.) and was considered a suitable reference gene [62]. Similarly, *Actin* showed high expression stability across multiple tissues in barley (*Hordeum vulgare* L.) and oat (*Avena sativa* L.) [63], and *ACT2* performed optimally under abiotic stress conditions in blackgram (*Vigna mungo* L.) [64]. The eukaryotic translation factor *eIF5A* plays an essential role in translation elongation and general translational regulation, particularly in facilitating the synthesis of proteins containing specific amino acid motifs, and is required for maintaining basal translational activity in eukaryotic cells [65,66]. Previous studies have demonstrated that *eIF5A* exhibits favorable amplification specificity and linearity (R^2^ > 0.98) across different fruit developmental stages and storage conditions in longan (*Dimocarpus longan* Lour.), highlighting its potential as a reference gene [67]. In addition, a systematic evaluation of commonly used reference genes in *Iris lactea* var. *chinensis* under heavy metal and salt stress conditions revealed that *eIF5A* maintained high expression stability and was identified as one of the most suitable reference genes [68]. Collectively, these studies provide functional and experimental evidence supporting the relatively consistent expression patterns of *ACT-2* and *eIF5A-1* observed in the present study. Integrating transcriptome analysis with RT-qPCR validation, *ACT-2* and *eIF5A-1* consistently exhibited high expression stability across different tissues and multiple stress conditions and were therefore ultimately selected as the optimal candidate reference genes. It should be noted that several commonly used reference genes, such as *GAPDH*, *Tubulin*, and *EF1α*, were not retained in this study because reference gene suitability is highly species- and condition-dependent, and genes widely used in other plant species may exhibit variable expression stability in a different biological context. To provide supplementary biological context, the evolutionary relationships and predicted protein structural features of these two genes were briefly examined. Phylogenetic analysis demonstrated that *ACT-2* and *eIF5A-1* possess clear orthologous relationships in *Arabidopsis thaliana* and *Oryza sativa* and form distinct clades in the phylogenetic trees, indicating a high degree of evolutionary conservation. Meanwhile, AlphaFold-based predictions revealed that both *ACT-2* and *eIF5A-1* proteins adopt well-folded, structurally complete three-dimensional conformations with high overall model confidence, reflecting their evolutionary conservation as fundamental functional proteins, but not implying a direct relationship with transcriptional expression stability.

## 4. Materials and Methods

### 4.1. Plant Material, Growth Conditions and Stress Treatments

Wild-type purslane (*Portulaca oleracea* L.) plants used in this study were collected and maintained by our laboratory from Guangzhou, Guangdong Province, China. Two weeks after germination, uniformly sized seedlings were selected and hydroponically cultured in half-strength Hoagland’s nutrient solution for an additional two weeks, with six seedlings per container. The nutrient solution was renewed weekly. Subsequently, seedlings were subjected to the following treatments: control (CK), 120 mM NaCl, high-temperature stress, low-temperature stress, mechanical damage (MD), 1 μM jasmonic acid (JA), 1 μM abscisic acid (ABA), and 8% (*w*/*v*) PEG6000. High- and low-temperature treatments were conducted in climate chambers set at 42 °C and 12 °C, respectively. All other treatments were performed in a controlled growth chamber maintained at 27 °C with 70% relative humidity. Plants were grown under a 14 h light/10 h dark photoperiod with a light intensity of approximately 20,000 lux. After 24 h of treatment, leaf samples were collected from each group, immediately frozen in liquid nitrogen, and stored at −80 °C for subsequent RNA extraction and gene expression analysis.

### 4.2. Total RNA Extraction and cDNA Synthesis

Total RNA was extracted from leaf samples using Cinzol reagent (CINOTOHI, Changsha, China) according to the manufacturer’s instructions. The concentration and purity of RNA were determined using a NanoDrop One spectrophotometer (Thermo Fisher Scientific, Waltham, MA, USA), and RNA integrity was assessed by electrophoresis on a 1% (*w*/*v*) agarose gel. To eliminate potential genomic DNA contamination, the extracted RNA was treated with DNase I (Solarbio, Beijing, China) following the manufacturer’s protocol. cDNA was synthesized from 1μg of total RNA using the PrimeScript™ RT Master Mix (Perfect Real Time) kit (Takara, Kyoto, Japan) according to the manufacturer’s instructions. The resulting cDNA was diluted fivefold with nuclease-free water and stored at −20 °C until further use in RT-qPCR analysis.

### 4.3. Primer Design and Analysis for RT-qPCR

Candidate reference genes were selected based on our laboratory-assembled and annotated purslane genome, together with transcriptome datasets derived from multiple tissues. Genes showing relatively stable expression across root, stem, leaf, and flower tissues were screened, and ten candidate reference genes representing six housekeeping gene families were ultimately selected, including *Actin*, *protein phosphatase 2A* (*PP2A*), *cyclophilin* (*CYP*), *ubiquitin*, *eukaryotic translation initiation factor 4A* (*eIF4A*), and *eukaryotic translation initiation factor 5A* (*eIF5A*).

RT-qPCR primer pairs were designed according to standard criteria to ensure optimal amplification performance, including an amplicon length of 100–250 bp, GC content of 40–60%, primer length of 20–25 bp, and a melting temperature difference of less than 3 °C between forward and reverse primers. Primer sequence information is provided in Table 1. Gene structure diagrams were generated based on genomic annotation files using TBtools (v2.376) to guide primer placement. To minimize genomic DNA amplification, primers were designed to span exon–exon junctions whenever possible. In addition, primer binding sites were deliberately avoided within untranslated regions (UTRs) to reduce potential transcript isoform variation. Primer design was performed using SnapGene (6.0.2) software, and primer specificity was further evaluated in silico using TBtools by aligning primer sequences against the purslane genome. For gene families with multiple predicted copies, primers were designed to target conserved coding regions shared among gene copies whenever possible, in order to reduce copy-specific amplification bias. Candidate genes that failed to produce clear and reproducible single amplicons in conventional PCR assays were excluded from subsequent analyses based on amplification performance under the tested experimental conditions. The final amplicon sizes of the candidate reference genes ranged from 152 bp (*ACT-2*) to 205 bp (*Ubi-1*), as shown in Table 2. The coding sequences (CDSs) of all selected candidate reference genes are provided in the Supplementary Information (Figure S2).

### 4.4. PCR and RT-qPCR Analysis of Gene Expression

To evaluate the expression characteristics of candidate reference genes, cycle threshold (Ct) values were obtained from five different tissues (seed, root, stem, leaf, and flower) and under seven stress treatments, including NaCl, high temperature (42 °C), low temperature (12 °C), mechanical damage (MD), PEG treatment, jasmonic acid (JA), and abscisic acid (ABA). Ct values generated from RT-qPCR assays were subsequently used as input data for expression stability analysis. All experiments were performed with three technical replicates to ensure data reliability.

Primer specificity was first validated by conventional PCR using cDNA templates derived from the five tissues. Each 10 μL PCR reaction contained 8 μL of 1.1 × S4 Fidelity PCR Mix, 0.5 μL each of forward and reverse primers, and 1 μL of cDNA template. The PCR amplification program consisted of an initial denaturation at 95 °C for 2 min, followed by 35 cycles of 98 °C for 30 s, 57 °C for 15 s, and 72 °C for 30 s, with a final extension at 72 °C for 5 min. Amplified products were examined by electrophoresis on a 1% (*w*/*v*) agarose gel stained with ethidium bromide to confirm primer specificity. RT-qPCR was performed using an ABI 7500 Real-Time PCR System. Each 10 μL reaction mixture contained 5 μL of 2 × SYBR Green qPCR Premix, 0.4 μL each of forward and reverse primers, 1 μL of cDNA template (diluted 1:5), and 3.2 μL of RNase-free water. The thermal cycling conditions were as follows: 95 °C for 30 s, followed by 40 cycles of 95 °C for 10 s, 57 °C for 30 s, and 72 °C for 34 s. A melting curve analysis was conducted immediately after amplification using the following program: 95 °C for 15 s, 60 °C for 60 s, 95 °C for 30 s, and 60 °C for 15 s. All reactions were conducted in three technical replicates, and raw Ct values were compiled for subsequent analysis.

### 4.5. Bioinformatic Characterization of Candidate Reference Genes

To provide complementary genomic and structural information for the candidate reference genes, a series of bioinformatic analyses were performed. Chromosomal locations and basic physicochemical properties of the candidate reference genes were obtained based on the annotated purslane genome using TBtools (v2.376), with gene positions displayed as points scaled to chromosome length. Conserved domain analysis was conducted using the Conserved Domain Database (CDD) provided by NCBI and the identified conserved domains were visualized using TBtools (https://www.ncbi.nlm.nih.gov/) (accessed on 10 November 2025). Promoter regions were defined as the 2000 bp upstream sequences from the translation start site and extracted from the purslane genome. Cis-acting elements regulatory elements within these promoter regions were predicted using the PlantCARE database, and the distribution of cis-acting elements was illustrated with Tbtools (https://bioinformatics.psb.ugent.be/webtools/plantcare/html/) (accessed on 28 November 2025).

To explore the evolutionary relationships of the finally selected reference genes, homologous sequences were retrieved from *Arabidopsis thaliana* L. (https://plants.ensembl.org/index.html) and *Oryza sativa* L. (https://rice.uga.edu/download_osa1r7.shtml) genome databases (accessed on 17 November 2025). The two selected purslane reference genes were used as queries to identify homologs at the whole-genome level using BLASTP (BLAST+ 2.16.0) and hidden Markov model (HMM) based searches implemented in TBtools and Pfam (https://pfam.xfam.org/) (accessed on 20 November 2025). The identified homologous protein sequences were subsequently used to construct phylogenetic trees using MEGA version 12. In addition, to further describe the structural features of the two selected reference proteins, three-dimensional (3D) protein structure models were predicted using the AlphaFold (https://alphafoldserver.com/) (accessed on 6 January 2026) and visualized with PyMOL (3.1.6.1).

## 5. Conclusions

In this study, candidate reference genes for *Portulaca oleracea* were systematically screened and evaluated based on transcriptome data across multiple tissues and various abiotic stress conditions. By integrating bioinformatic analyses with conventional PCR, RT-qPCR, and dilution curve assays, *ACT-2* and *eIF5A-1* were identified and proposed as candidate reference genes with application potential under the experimental conditions examined in this study. The results indicated that these two genes exhibited relatively consistent expression characteristics across different tissues and under multiple abiotic stress treatments, together with favorable amplification performance. This study provides a practical reference for gene expression normalization in purslane and offers a methodological framework for the systematic identification of reference genes in other non-model plant species.

## Figures and Tables

**Table 1:** Physicochemical properties of candidate reference genes in *Portulaca oleracea* L.

Gene Name	Gene ID	Annotation	Protein Size/aa	MW/ kDa	pI	Instability Index	Aliphatic Index	GRAVY
*ACT-1*	*PolA03G003200.1*	Actin family protein	389	44.28	5.43	33.14	91.39	−0.209
*ACT-2*	*PolB12G012440.1*	Actin family protein	139	16.2	5.49	44.66	68.06	−0.54
*PP2A-1*	*PolB10G002880.1*	Protein phosphatase 2A catalytic subunit	308	35.16	4.97	37.47	83.54	−0.298
*PP2A-2*	*PolA05G027100.1*	Protein phosphatase 2A catalytic subunit	398	45.03	5.21	47.12	67.29	−0.821
*CYP-1*	*PolA02G030460.1*	Cyclophilin family protein	684	75.31	11.05	89.12	35.1	−1.462
*CYP-2*	*PolB13G003700.1*	Cyclophilin family protein	828	93.96	11.72	130.5	38.39	−1.51
*eIF4A-1*	*PolA05G013700.1*	Eukaryotic translation initiation factor 4A	625	67.77	6.68	62.9	53.06	−0.964
*Ubi-1*	*PolA01G008400.1*	Ubiquitin family protein	3750	407.78	5	49.78	90.79	−0.229
*Ubi-2*	*PolA05G021080.1*	Ubiquitin family protein	793	87.37	9.38	47.02	67.99	−0.613
*eIF5A-1*	*PolA08G004380.1*	Eukaryotic translation initiation factor 5A	159	17.41	5.6	28.11	79.06	−0.534

MW, molecular weight; pI, Theoretical isoelectric point; GRAVY, grand average of hydropathicity.

**Table 2:** RT-qPCR Primers for Reference and Validation Genes.

Gene ID	Primer Melting Temperature (Tm)	Primer Sequence(5′ to 3′)	PCR Product Size (bp)
*PolA03G003200.1*	56 °C 55 °C	AAGCCTATGCTACGGTATGAAG CTTCAATTCATTGAAGAATGCGTG	183
*PolB12G012440.1*	55 °C 57 °C	CTGAGAATGAGTGTCGTTATGC CTTGAATCGGTCCTTAGAGCTT	152
*PolB10G002880.1*	56 °C 56 °C	CGATTTGATCGAGTTGTTTCGAA TTTCCTCGGAGGATTGTAATCC	162
*PolA05G027100.1*	55 °C 58 °C	GAGCAGTGCGAAGATATGATTAG TGCTTGCGCAGTCTTGATAA	170
*PolA02G030460.1*	56 °C 57 °C	GAGAAAGGGATTGGAGCAAC CTCATCTGCAAGAAATGCCAAT	174
*PolB13G003700.1*	56 °C 56 °C	ATCAGATAGTAAAGGGCTCTGTG AGCCTCAAGTGTAAGGCTAAATAT	194
*PolA05G013700.1*	56 °C 56 °C	TGGAATCAGTTGAAGAGGAGAC AATTTCCTACCACCAAATGTTCG	172
*PolA01G008400.1*	56 °C 54 °C	GGACAACCAGTTGACATGGATAA GATTAGTCAATCTGTGACTGCT	205
*PolA05G021080.1*	57 °C 57 °C	TCTTCATCTAGAAAGGCAGAGGT TGCCACTTTGCAGATATGCT	187
*PolA08G004380.1*	60 °C 57 °C	ATCGTCATCAAGAATCGCCCTTG TCAGCTGATAATCAGTACGGTTG	184

## Data Availability

The original contributions presented in this study are included in the article/Supplementary Material. Further inquiries can be directed to the corresponding authors.

## Supplementary Materials

The following supporting information can be downloaded at https://www.mdpi.com/article/10.3390/ijms27052276/s1.
