# Supplementary material for: Screening and Stability Validation of RT-qPCR Reference Genes in Portulaca oleracea L. in Diverse Tissues and Under Abiotic Stress Conditions
Source: Int J Mol Sci. 2026 Feb 28;27(5):2276. doi: 10.3390/ijms27052276 (PMC12986012; doi:10.3390/ijms27052276)
Supplement: Supplementary file 1 [file ijms-27-02276-s001.zip › ijms-4138798-supplementary.pdf]

Screening and Stability Validation of RT-qPCR Reference Genes in *Portulaca oleracea* L. under Diverse Tissues and Abiotic Stresses

Jiahui Fang [1], Chenxin Fan [2], Jieshan Wang [3], Ming Yi [4], Ping Li [5], Mengyun Xu [6,*] and Jian Yan [7,*]

[1] Key Laboratory of Agro-Environment in the Tropics, Ministry of Agriculture and Rural Affairs, Guangdong Provincial Key Laboratory of Eco-Circular Agriculture, Guangdong Engineering Research Centre for Modern Eco Agriculture, College of Natural Resources and Environment, South China Agricultural University, Guangzhou 510642, China; fangjiahui1109@gmail.com (J. F.); 2901327619@qq.com (C. F.); 924216522@qq.com (W. J.); ymxjy20210116@163.com (Y. M.); liping2016@scau.edu.cn (L. P.);

* Correspondence: yanjian78@scau.edu.cn; xumengyun121018@scau.edu.cn

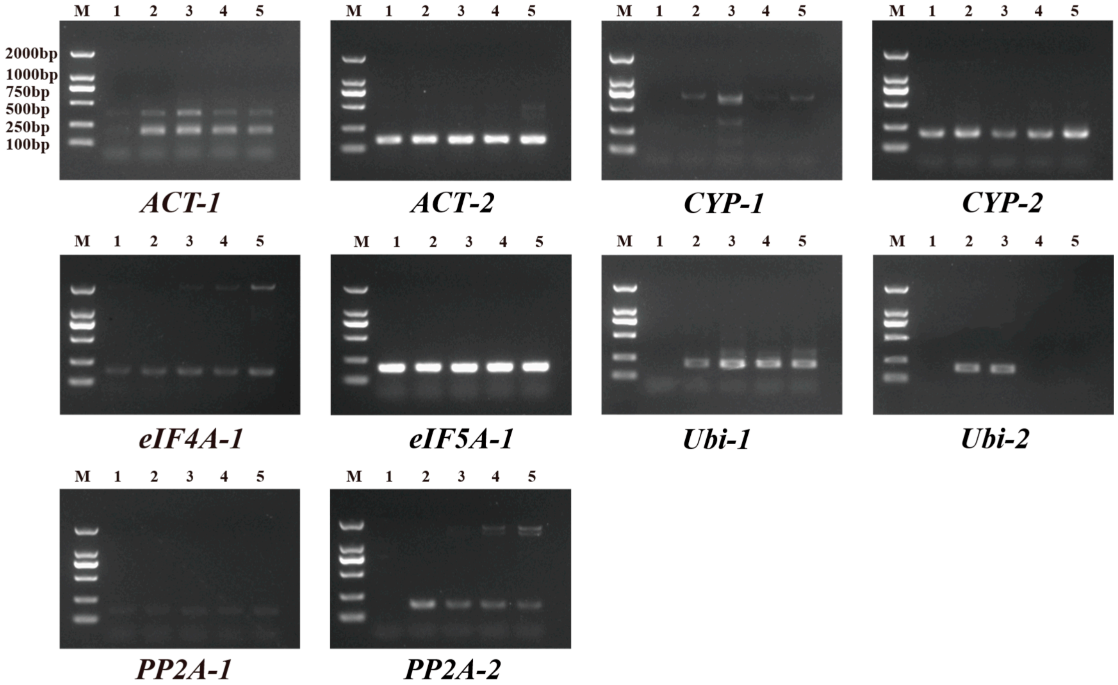

Figure S1. Uncropped agarose gel electrophoresis images of conventional PCR products for candidate reference genes. Conventional PCR was performed using cDNA from different *Portulaca oleracea* tissues. All gel images are shown in their original, uncropped form. M, DNA marker (DL2000); band sizes (top to bottom): 2000, 1000, 750, 500, 250, and 100 bp. Lanes 1–5 correspond to cDNA derived from seed, stem, flower, leaf, and root, respectively. Gene names are indicated below each gel panel and correspond to the candidate reference genes listed in Table 1.

>PolA03G003200.1 gene name=ACT-1

ATGGACAACAAGAATGTCGTCGTCTGCGACAATGGCACTGGGTATGTTAAGTGTGGCTTTGCTG
GAGAGAATTTTCCCACCTCAGTTTTCCCTTGTTTGGTGGGGAAGCCTATGCTACGGTATGAAGAA
TCACTAATGGAACAAGAATTGAAGGACATTGTTGTTGGCGAGCCTTGTTTGAAGTATCGAAACC
AACTAGACATCTCTTACCCTGTCAATAATGGAATAATTCAAAACTGGGATGACATGCTTCACGT
ATGGGACCACGCATTCTTCAATGAATTGAAGATAGATCCAGCAGAGTGTAAGATCCTTCTCACA
GACCCCCCACTTAATCCATCAAAGAACCGTGAGCAAATGGTTGAGACAATGTTCGAGAGATAC
AATTTTTCTGGCATCTTTATCCAAATCCAGGCAGTCCTAACACTATATGCTCAAGGTTTGCTAAC
TGGATTAGTCATTGACTCTGGAGATGGCGTAACGCATGTGGTTCCTGTTGTTGATGGTTACTCAT
TTCCACATCTTACAAAACGAATGAATGTGGCAGGGAGACATATAACATCTTATCTTGTGGACTT
GCTCACGCGGAGAGGGTATGCGATGAATAGGGCTGCTGATTTTGAAACTGTTAGGCAAATCAA
AGAGAAGCTCTGCTACGTAAGTTATGATTATAAACGAGAGTATCAGTTGGGACTTGAAACCAC
CATTCTGATGAAGAAATACACTCTGCCTGATGGAAGGGTTATCAAAGTTGGCACCGAGCGGTTC
CAAGCTCCAGAAGCTCTCTTCACCCCAGACCTCATTGATGTTGAAGGCGATGGAATGGCTGATA
TGGTATTCCGCTGCATCCAGGAAATGGATATTGATAACCGCATGATGCTTTATCAACATATTGTT
TTAAGTGGAGGGAGCACCATGTACCCTGGACTACCAAGCCGGCTGGAAAAGGAAATTTTGGAT
CGATATCTCGATGTTGTGCTGAAGGGAAACAAAGATGGGTTAAAGAAGCTGAGACTAAGGATA
GAGGACCCTCCACGAAGAAAGCACATGGTTTACCTTGGAGGTGCAGTTCTTGCTGGAATTATGA
AGGATGCACCGGAGTTCTGGATTAGCCGGGAAGATTATCTGGAGGAAGGTCTTGGCTGCCTCTC
CAAGTGTGGACCAGCTTGA

>PolB12G012440.1 gene name=ACT-2

ATGTCGAACTCAGCATCAGGTATGGCCGTGCACGACGACTGCAAGCTAAAATTCTTGGAACTG
AAGCAAAAACGCACTTTCCGATTTATTGTTTATAAGATTGATGAGAAGGCTAAGGAGGTGGTGG
TTGAAAAGCTTGGAGAACCAGCTCAAACTTATGAAGATTTCACTGCATGTCTTCCTGAGAATGA
GTGTCGTTATGCTGTTTATGATTTTGACTTTGTGACTGAGGAAAATTGCCAGAAGAGCAGAATCT
TCTTCATTGCCTGGTCTCCCGACACAGCAAGGGTGAGGAGCAAAATGATTTACGCAAGCTCTAA
GGACCGATTCAAGAGGGAATTGGATGGCATTCAGGTGGAGCTGCAGGCTACCGATCCAACTGA
GATGGGACTCGATGTTTTCAAGGACCGAGCCCACTGA

>PolB10G002880.1 gene name=PP2A-1

ATGCCGTCTCATGCGGATCTGGATCGGCAGATCGATCAATTGATGGAATGCAAACCGTTGTCGG
AATCGGAGGTGAAGACGCTTTGCGATAAAGCGAGGGCGATTTTGGTCGAGGAATGGAATGTAC
AGCCAGTGAAGTGTCCGGTGACTGTTTGCGGGGACATTCATGGCCAATTTTACGATTTGATCGA
GTTGTTTCGAATTGGCGGGAGGGCTCCTGATACTAATTATCTTTTCATGGGCGACTATGTAGACC
GTGGATACTATTCAGTAGAGACTGTCTCTCTTCTGGTTGCTCTAAAGGTTCGTTACAGAGACCGG
ATTACAATCCTCCGAGGAAATCATGAAAGCCGTCAAATTACTCAAGTGTATGGGTTTTACGATG
AATGCCTGAGGAAGTATGGAAATGCAAATGTCTGGAAGCATTTCACTGATCTGTTCGACTACTT
ACCTCTCACAGCTCTCATTGAGAGTCAGGTTTTCTGCTTGCATGGAGGCCTTTCACCATCTTTGG
ATACTTTGGACAATATTCGTGCCTTGGATCGCATACAAGAGGTTCCTCATGAAGGACCTATGTGT
GATCTCTTGTGGTCTGATCCTGATGATCGGTGTGGTTGGGGAATTTCCCCTCGTGGAGCTGGATA
TACCTTTGGACAGGACATAGCAGCTTCATTCAACCATACCAATGGACTTACTCTCATCTCTAGA
GCCCATCAGCTTGTGATGGAAGGTTACAATTGGTGTCAGGTTAGTGAGAAAAACGTTGTTACAG
TTTTTAGCGCTCCTAATTACTGTTATCGATGTGGGAATATGGCCGCCATACTTGAGATTGGAGAG

AATATGGATCAGAACTTCCTGCAATTCGACCCTGCACCTCGGCAGATTGAACCCGACACCACTC
GCAAGACCCCCGACTATTTTTTGTGA

>PolA05G027100.1 gene name=PP2A-2
ATGGGGGAATCGAAGATGGCGGAGATGACGCTGCCTTCTCTGTTCGAACAAGCTCGCAAGATC
CACCAATTAGCATCTGATTCGTCCGCTGATCAGGAGACTATAAAGAAGGGCTGTCAGGTGCTGG
AGCAGTGCGAAGATATGATTAGCAAGCTCGGATTGTTTTCTACAAATGAAACTAAAGACGATA
TTAGCACTGCTAATCTCAAGTATCTACTGGTTCCATATTATTTAGGCGAGCTAACAGAAAAGGTT
CAACAAGATGACAGGATGCAAATTATCAAGACTGCGCAAGCAAAACTGAAGGAATTCCTTTCA
TTTTGTGAATCCATGGAGCTTCTTCCTCAAGAAGAGTTGGAATCTTCTGGTGGTACTTTTGCTGA
CAAAAGGGCCAGAAAGATTGCTCGCTTTAAACGGCAAAAAGCGGCAGAGTCCAAATTGTTGGA
GATTAAGGAACGCAGGGAACGTCGAGGGCGTTCAACAAGAGCAGCAGCATTGTCCACACCGG
TGGAGGCAGGGGAAGAAGATGTGGTGGATGATGATGGGGAAGAAGAAAGGGAGGCATGGCTC
ATTACAATATCATTGGCGATTTGCAAGACCTTGGATTTGATTGAAATGCTGAAGAAAGAGGAAG
AGATGCTCTCTGCCGTGAAGGAGAGACAAGCACAGGATGGCGGCAAAGAATTGACTCAGTCTC
TTTTAGATGAGCGAACAAGGAAGGCAGAGTCATGGCATCGTGATGCTGCAGCTCGTGCTCAAT
ATACAAAACCAGCACAGCCCATAACATGCGCCACATTTGCTCAAGATGTGTTGGAAGGAAGAG
CAAATGTGTCTCAGGCACATGAACACAAACATCAGCCTCTCATATTTGGGCCTGCAAGTGCTGT
GGGTGGAAATCTTACAACCGAAAGAGAGCGAATGGTAGCCCAAGTTTTCCAACCAAGCCATAG
GATGCCAACCATGAGCATAGAGGAAGCCGGACTGAAGGAGATGGAAATGATGAACAAATGGC
AAGAAAGAACCAAAAAATTTATCGAAGAATCAAACTCATCTTGGCACAGGGATCCTCCAAAAC
CAGGCCCTCCCGAGGAGGATGAAGATGATGACGCCGCAGTTGAGAGGGCTCGAGCATTCGATG
ACTGGAAGGATGAGAATCCCCGTGGTGCAGGCAACAAGAAGCTCACTCCCTGTGGTTAA

>PolA02G030460.1 gene name=CYP-1
ATGGCGAAGAAAAAGAATCCTCATGTATTTCTAGATGTTTCTATTGGTGGGGATCCAACCGAAA
GAATTGTTATTGAGCTTTTCTCAGATGTCGTTCCTAAAACTGCAGAAAACTTCCGGGCTCTATGC
ACTGGTGAGAAAGGGATTGGAGCAACGACAGGAAAACCTCTGCACTACAAAGGTTCAAATTTC
CACCGCATAATTAAAGGATTTATGGCTCAGGGAGGTGATTTTTCGAAGGGAGATGGAACCGGT
GGAGAGAGTATCTATGGAGGCAAGTTTGCAGCATTGGCATTTCTTGCAGATGAGAATTTTAAAC
TGGAACATAATGGACCGGGACTCCTCTCCATGGCAAACTGTGGCCCTAATACGAATGGGTCCC
AGTTTTTCATAACATTCAAGCGTACTCCGCATCTCGATGGGAAACATATTGTGTTTGGGAAGGTT
GTGCAGGGGATGGACATTGTGAAAAAATTGGAACAGGTTGGCTCTTCTGACGGGCGGCCTCTTA
AACCAGTCAAAGTTGTCGACTGTGGTGAAGTTGCTGCTGGTAAACCTGAGTCTGCAGTCCGACC
TGAGAAAGGGAAAGTTAAGAAATCACGGAAAGCCAGTAGCTCAGATGATAGTTCAGATGGCA
AAGGCCGAGGTAGGCGGCGCAAAAGCTCTAATGACAGAAGGAAAAAGAAGAGGAGATACTCT
TCATCGAACTCTTACAGCAGTTCTGGATCAGACTCTGATTCTGAATCCTACACTTCGGACTCAGA
CACTTATTCTGATTCAGAGTCGGATGATTCACAATCTGACTCGAGTTCCGACAGTGAAGATCGG
CGCAGGAAGAGAAAGAGACCCGGTAAAAGCTCTAAATCTAAGCGAGTAGCGAAAAAGTCAGG
ACGAAGTGAAAAGAAGAAAGGCCGTCATAATGGAAGAGCTAGGCGCAAGTCAAAAAGGAGTT
CTGGGAGCTCAAGTGATACTGGAAGTGAGACACCAAGCAGTTATGAGGATGAAAAGTCAAGA
CGTCGCCATTCGTCAAAGACGGAGAAGAAATTATCTGTTGAAAAGCCGCTGAAGGATCAAAAC
CAGAAGAAAGACAGTGATTTGCCTGCGAAGGCTGAAGGTAGTTTGAACAATGGACGTGGCAAG
GAATCTAATTCTGATAAAATAGCGGATCCACTTTCCTCGGATGAGGGTGCAGCTAAGTCCAGGG

GCCGATCTCCAAGCCCAAAAACAAGTCAAAAGATTAGTCCTAGCCCAAGCCCAAGCCCAAGG
AGAAGAAGTCCTAGTGTGAGCCCCGATAGAAACCGGAAGAGGAGTCCCAAGCCCGAGGCTGA
AGCCATTGCACCTCCGCCCGCTTCCAACGACCAAGGGCAGCAAGAGGTGTCCAAAAACCCCGA
TGAAGACAACGGTGGGGCCCCCAAGCGCGTCAGGAAAGGACGGGGATTCACTGAGAAGTTTG
CCTTTGCACGCCGGTACAAGACCCCTTCTCCCGAGCGTTCACCCCCACGCAGGCCGTACAACAA
TTATGGTGGTAGGGGCTACTATGGAAGGAATCGGGATAGATTTTCAAGCTACAGAGGTTACTCA
GATCGCTCGCCATACAGGCGCTACAACAGGAGCCCTCCAAGGAGGAGATCCCCGAGAGGCAG
GAGAAGCCGAAGCCTCTCACGAAGCCCTAGTGATAACCGAGGCAGTGGAAGGAACCGAACCC
GGTCCCGGTCTCCGGCTCGGAGTCCCAGCCCAAGGGACAGGCGTCCTCCAATGAGTGACCGGTT
AAGGTCCCGTCTCGGGCCTCGTGCAGATGACCGCCGCTCTCCCCCACCCCGAGCCAGAAGGTC
AAGGTCCAGGTCCAGGTCCAGGTCCAGGTCCAAGTCCAGGTCCAGGTCAAGGTCAAGGGGAAG
AAGCGTATCTCGATCGAGGTCTCCTCCTGCCGCTGATGCCCCAAAGAGACGAGGCAAAAGAGG
GTCTTCATCTCCGAGGTCGACTTCAAGCTCACCCGTAGTAGGGCAAAAGGGTCTGGTAGCCTAC
GGCGATCCCAGTCCTGATGGTGGTTCTAGATAG

>PolB13G003700.1 gene name=CYP-2
ATGGCTAAGAAGAAAAACCCATTTGTTTTTTTGGATGTCTCTATTGATGGAGACCCTTATGAAAG
AATGATTTTTGAGCTCTTTTCTGATGTTGTTCCAAAGACTGCAGAGAACTTTCGTGCATTATGTAC
AGGGGAGAAGGGAGTTGGCCCCAAGCTGAGGAAACCTTTACACTACAAAGGAACTTTTTTCCA
TCAGATAGTAAAGGGCTCTGTGGCGCAGGCTGGCGATCTTCTCAGACAGGAGGGCATGGCTGG
AGAAAGTATATATGGGGATAAATTTCCTGATGAGCTGTCCCATCTAAAGCATGACGAGCCTGGT
CTGTTGTCAATGTCGGTTGCTGATCGTGATGTGCGTGGATCAATATTTAGCCTTACACTTGAGGC
TAACCATAATCTCGATAGGAAGAATGTAGTGTTCGGTAAGCTTGTACATGGATATGATGTGTTG
AAGAAAATTGAGGATGCGGGTGATGAGGAGGGGAAACCAGCTGTTACAGTAAAGATAGTCAA
CTCTGGTGAATGTAGTGAGAGGGATAAGAAGAAACTTAGCAAGAAGTTGGGAAAGGCGGGTG
TAGTTGATGCTAATAATCATGGAACACGACATAAGGGGAAGCATAAGAAATCATCCAGAGACA
GGAGAAAGAGGAGAAGAAAATATTATACGTCTGAATCCGAGAGTTCCTCTGACAGTGAATTGG
ATAGTACTGACTCTGATACTGAATCTGAAACCGAGAGTTCCTCATCAGACATCAGCTCCTCCAG
TGATGACAGGCGCAGGAAAAGAAAGAGATCGTCAAGGCGCGATAAGTACAGACGTGGAAAGC
GTAGCAGACGGCGTGATAAGAAACGCAGAAGGCATGATAAGCGTGCGAGACGCAAGTCAAAG
AGGTCATCGGAAAGTCTCAGTGATACTGATAGTGATAGCAAAACTAATAGTAGCTCTGAAGAC
GAGGTCAATGATAAGGAACATGCTCAGAAGCACAAAGAAGGTTCACAGAAAGCTGAGGGTGT
AACTGCTGGTAATAGCCACAAAAGAGGTGAAGCTTCTGCAAAGTTGGATGATGAGCACATCAG
ATCTCCCAGGGAAAATGGAAACCAGCGAAGCAATGATTTTGAAAGAGACGCAAGATATGGTA
GAAGCGAAGACAGGCAACCGGATGTGGTGGAGGACCACCCTGGCAAACCTAGGAGCCGGAGT
GCGAGCCCCAAACGGGCTATGAGTAAGAGTATGAGCATTAGTCCAAGGAGCATGAGCAGGAG
CCGCAGTCCAACCCCACGGAAGAGTGTGAGCAGAAGTCCAAGTGTTAGTAATCCAAAAGCTGC
AGAGAGAATTCGCGGTACCAGCGAGAGTCCACTAAGGAGTGGCAGTGCTAGAAGTGTCAGCA
GAAGCCCAGCGAAGGCGCGGTCTTGGAGGAATTCTAGCATAAGTCCTGCCAGGTCCCCTCCTC
AGAGAAGCCCTAGTGGGAGTCCCCCTAGAACCTCATCTCGGAGATCTGGAAGAAGTCCTGGAA
GATTTGGTAGAAGAAGTAGCCCTAGTCCCATTAGGTCATCTTATAGAAGTCCTAGCCCTATAAG
ATCTTCACGTAGAAGCCCTAGTCCTATAAGATCTTCACGTAGAAGCCCGAGCCCTATAAGATCT
TCGCGTAGAAGCCCGAGTCCCATAAGATCTTCGCGTAGAAGCCCGAGCCCTGTAAGATCTTCAC
GTAGAAGCCCGAGTCCTGTAAGATCTTCACGTAGAAGCATAAGTAGAAGTTCAGGTCGTGCCC

CATCAAGGAGAGTTGCAAGCCGAAGTCCAATTAGATCTCCAAGGAGGAGCTATCGCCGGAGCT
ACTCTAGAAGTCCTAGCCGTAGGAGCCCTCCTTCCATTCGGCGTAGGAGTGTGTCCAGAAGTGT
GTCTCCTGAAGGCTCACCTAAACGTATTAGAAGGGGGAGAGGTTTCAGCGATAGATTCTCTTAT
GCTAGAAGATACAGGACCCCGAGTCCAGAACGTTATCGCTATGGGGGTCGTGACAGGTATCGT
CTCATATATCCAAGTTATAGGAGGTACTCTGATCGTTCTCCAAGGCGCTACAGAGGCTCAAGAT
CTCCTATAAGATACAGAAACAGAAGAAGCAGATCTCGAAGCAGGAGTGTGTCTCGAAGCCCGC
CCTATCGCCGCCGCCGTTACAGCCGCAGCCCTATCCGTAGCCGTTCGCCAGTTGACGCTCCAAG
ATACCGTCCGTCTCCTCGAGCAGAAAAACGGCCCTCTGCTAGCCGATCAAGGTCAAGGTCAAG
GTCAAGGTCATCAGGATCTAGGTCCTCTAGAGGCTCCCCACCTCCAAGGAAGGCTAGCCGGTC
ACGGTCGAGGTCAAGGTCAAGGTCAAGGTCAAGGTCAAGGTCAAGGTCCAAGTCGAGATCAA
GGTCAAGGTCAAGGTCAAGGTCATCTTCTGGGAGTCCGGCAGGAAAGAGGGGGCTAGTCTCAT
ACGGTGATGGCTCTCCAGATTAG

>PolA05G013700.1 gene name=eIF4A
ATGGCCAGCGATGGCGGAGGAGGAGAGTCCGAGTACGAGAGTGATCCTGAAGAGACCAAAAT
ATCGTTGAAGATGCGAAGGAGAGAGGCAAGTGACGATGAGGATGGTGAAGAATTGCATCATA
GGGCTGGGATCGCTTATTCCGGGGGTGAATCCGACGGTCAGGGTGGCGCCGCTGAGTATGACG
AAGAAGAGGATTACTATGATGAGCAAGAGGAAGAATTGGCTGACGGCGACGGCGAGGATGTG
GAGAAAATAGAAGTTTGCGGTGCAGAGGAGGAGGACATGGCCATGGTGAGAGAGGTGGAATC
AGTTGAAGAGGAGACTGAGAGTCGAAACCATCAGGGCGAGGGTCAACAGACGAAGGAGAGC
GAGCCGTTTGCCGTTCCTACGTCTGGTGTTTTTACATGCACGATGATCGTTTTCGGGACAATGCT
GCTGCTAGACACAGGCGAACATTTGGTGGTAGGAAATTATGGGAATCAAAAGATGACAGGAA
ATGGGGTCACGACAAATTTGAGGAGTTGAACATCCAGGACGTGCATTATGAAGAGGGGAGAA
GGTCGTCAAAAGGATCCTATAGAGGTCGTGGCAAAGCTAGAGGTATAGAACGCGGTGATGCTC
GACGAAACAGGTCAAGAGGATACAGTGGAAACAACATCCAGAACACTGCAACTAAAGATGTG
AAAGGAAGAGGTCTTAGAGGATATCAACCCTCAATGAAGAATAAGGGTGAAGCTCTTCCACCA
CAAAACAAACAGTCAGCGAAGTTGCCTGACAAAATTTCACATGTTGGATCGGGGAGATTTTCTG
GCTCAACAACCAACCCAAATGAAGCGCATCAATCAGTACCTGCTAAAAAAGTTTCCTCCAACT
TGAGCTCTGCATCACCTCCTTTTTATCCATCTGTTTCGTCCAGTAAGGATGTTACTAAGATCCAA
AAGGAAAGAGAATGGGGAAATCATAACAAAGGCATCAGCCCCCCAGTCACCGATAACAGTTT
TCCTATGCCACACTCAAATTCTTTGTTGCGAGGAAAGAATGTTGTCGGGTCTGTTGGCATAGACA
AACTGAATATTAATGACTCCATGTCTCCTGTTGGTGTGAGGCCGTTAAATAATGTGCATCTGCTT
CCTGCATCATCAGTGTCAAGTATTAATCACATGCTCCCATCTAGAGGGGATGGGAGGGGCATGA
CAGTGCGTGGGCCACCCATGTTTCTGCCTTCTCAAACAAATAACATAGTTGAAAAAGCTTCTGT
ACCAGCAACTCAAACTCTGCAGAAAAGCCCACTTCAAAATCGACCTCAGCCTTCTCTACAGTCA
TCAGTTCATCCTCTGGGTCAGTGTCCTCTTGGTGTCTCTCAAGGATCTTCTCCACCTAAAGCAGG
AAAAGAAACTGTCCAGGGAGCTGGAAGGGCCTCACTAGTGCATGGCGGTGCAAAAGTCAGGG
GATCCACTGGAAATATGGTTGCTGGTCAAGGTGATAAGAAGCTTCCAGCTTTCTTTCCAGTTATG
CAATTTCGGGGTCAGCATCCTGGTGGCATTGGAGTTCCTGCGGTCGGAATGGCATTTCCTGGAT
ATGTTGCACAGCCAAATGGTTTGGGAAATTCTGAAATGACATGGCTACCTGTATTAGCAAGTGC
TGCAGGTGCTCTGGGTGCCACGTATTGTCCACCATATCTTCCCATTGATGGTGCTCATCAACCTC
GGCCAACAGGGAAAACATCTGCCTTGCCTGCCCCTAGCAAGGATAATACTCCTAAAAAGCCTA
ATGATGATCCAAAGCCTCAGCAAAGATCTGAGTTAGTGAATCATGAGATCAGTCAGAGACAGA
ATAAGCCTCGCAGATACTCAGAAATGAATTTTAGCCAATGA

>PolA01G008400.1 gene name=Ubi-1

ATGAAACTCAAGCGGAGAAGGGCTCCAGAAGTGCCTCCAAAGATACGGTCTTTCATCAATAAT
GTCATCTCGTCTCCCCTAGACAGTATTCAGGAGCCCTTGAAAAGTTTTGTATGGGACTTTGAGAA
GGGGGATTTCCATCACTGGGTGGATCTTTTTAACCATTTTGACTCATATTTTGAGAAGTATGTAA
AGCCTAGAAAAGATCTACGTCTTGAGGATGAATTTTTGGATAGTGATCCTCCATTCCCTAAAGA
AGCTGTTCTCCAAATCCTCCGCGTTACAAGGATTATTTGGAGAATTGCACAAATAAACATTTTT
TCAGTTCGTATGAGCATCTATCATCTTTACTAGCTTGTGCTGATGCCGATGTGGTTGAATCTAGTC
TGAAGACTTTAGCAACCTTTGTGAAGAAGACAGTTGGGAAATATACTATTAGAGATGCTGCTCT
GAGTTCAAGATTATTCGCTTTTGCTCAAGGTTGGGGTTGCAAGGAAGAAGGGCTTGGATTAACT
TCATGTGCAGTGGAAAATGGGTGTGACCCTATTGCTCATCAGCTAGGCTGCACCCTCCACTTTG
AATTCTATACTGTGGATGAATCTTGCAGTAGTTCTGAAGCTACACGAGGTCTACAAATCATTCAT
TTGCCTAATGTTAACCGCATCGAGGAGAATGATCTCAAGCTTTTGGACAAGTTAGTAAAAGAGT
ACAAAGTGCCTCCTAGTTTAAGATTTCCTTTATTTACTAGACTGAGGTTTGCTAGAGCTTTTGGTT
CCCTGGCTTCTCGGCAGCAGTATACATGCATCCGATTGTATGCTTTTGTGGTAGTCGTTCAAGCA
AGTAGCGATGCTGATGACTTGACATCTTTCTTTAACAATGAACCTGAATTCATCAATGAGTTGGT
CTCATTGTTAAGTCTCGAGGATGCAGTTCCAGAGAAAATACGAATTTTAAGCCTGCGATCATTA
GTTGCTCTCTGTCAAGATCGGTCTCGTCAGCCTACTGTACTCGCTGCTGTGACATCTGGTGGACA
TCGTGGAATTCTTTCCAGCCTCATGCAAAAGGCCATTGATTCTGTCATTAATGGTTCTTCGAAAT
GGTCGGTTGTTTTTGCTGAATCTCTGTTGTCACTTGTCACTGTTTTGGTCTCGTCATCATCTGGTTG
TTCTGCAATGCGTGAAGCTGGATTTATACCCACACTTTTGCCCCTTTTTAAAGATACAGATCCTC
AACACCTGCATTTAGTTGCGACTGCTGTGCACGTTCTCGAAGCTTTCATGGACTACAGCAATCC
AGCTGCTGCATTGTTCAGAGACCTTGGTGGTCTAGATGATACTATTTATCGCCTTAAGGTAGAAG
TATCTGTCATTGAAAGTGGTTCCAAGCAAACATCTGGTGACGTTGATGGAAGCAGGTTGGTCAG
TGACTTCGTTGCTAGTACATCTTCTGCCCTAGATGACTCACAACCTGTTTACTCAGAAGCATTGC
TTACTTCTCATAGACGTTTGTTGATGAAAGCATTGCTACGTGCTATATCACTAGGAACCTATGCT
CCTGGTACTACTGCTCGTATTTATGGTTCTGAGGAAAGTCTATTGCCCAAGTGCCTGTGCATCAT
ATTTCGAAGGGCCAAAGATTTTGGTGGCGGTGTTTTTTCTCTTGCTGCAACTGTTATGAGTGATCT
AATTCATAAGGACCCTACATGCTTTTCTGTCCTTGATGCAGCTGGTCTGCCCTCGGCCTTCATGG
ATGCTATAATACAAGGTGTTGTGTGCTCCGCAGAAGCCATAACTTGTATACCTCAGTGTCTTGAC
GCATTGTGTCTGAACAATAACGGTCTTCAGGCAGTCAAAGATCGCAATGCTCTGAGTTGTTTTGT
GAAAATATTCACTTCAAGACCATATTTACGTGCCCTTGCTGGGGATACTGCTGGGTCCTTGTCAA
GTGGCCTAGATGAACTTATGCGCCATGCATCTTCATTACGTGGGCCTGCAGTTGATATATTGATT
GAGATCTTGAACACAATTTTAAAACTTGGGTCTGCAGTTGAAGTGCCATCCCCATCTGTTGATTC
TTTATCCTCCCCAAATCCTGTTCCTATGGAGACGGATTCAGAAAGCAGGGAGGGGAAGGAATT
GTCAAAGATAGAAACTGCTGATGCGAATGATCTGTCTTCTGACTTATCTTCTGCAAATGTTGAGT
CTTACCTTCCAGACTATGTTAGCAATGCTGCTCGGCTTCTTGAGACTATTCTCCAGAATTCAGAT
GCATGTCGCCTATTCATTGAGAAGAAAGGAATTGAGGCTCTTCTAAAGATGTTTTCTTTGCCATT
ACTCCCTCTTTCAGCATCAGTAGGTCAATCTATTTCAATTGCTTTTAAAAACTTTTCTGCACAGCA
TTCGGCTTCCTTGGCAAGGGCTGTGTGTTCATTTTTGAGGGAGCATTTAACCACAACTGTTGAGC
TGTTAGATTCGATTGGAGGGACCCGTCTTACTGATCTGGAATCTGAGGAGCAGACGAAGGTCTT
GAAATGCCTTTCATGTTTGGAAGGCATCTTATCTCTTTCAAATTCCTTGTTGAAGGGGACTACAA
CAGTTGTCTCAGAGTTGGGGACTGGGGATGCTGATGTGTTGAAAGATATTGGCAGGGTATATAG
AGAAGTACTCTGGCAGATTTCTTTGCCTTCTGATTCCAAAGTGGAAGAGAAGCAAAATGTTGAC

ATGGAGACTGAAAATGCTGATGCAGCTGCTTCTAATGCTACTGGGAGAGAGAGCGACGATGAC
ACGGGCATCCCAGTTGTAAGATATATGAATCCTGTTTCGGTTAGAAGTGGTTCTCAGTCACAGT
GGGCTGGGGAGCATGATTTCCTGTCAGTAATTCGCTCCGGTGCGGGACTCAGTCGTCGTAATAG
ACATGGATTGACACGAATAAGAGGTGGGAGGACTGGTCGTCATCTTGATGCTTTTAACATTGAT
ACAGAATTATCTGTCAATGTAAATGAGGCTTCCACCTCTCTTGAACAGAAGAAAAAAAGTCCTG
ATATCCTTGCATCTGATATCCTTGCTAAGCTTTCTGGTACTGTGCGCTCGTTTTATGCAGCCCTCA
TAAAGAGCTTCAGTTCACCAAATCGTCGCAGATCTGATTCAGGGCCATTGAGTTCAGTTGCCAA
AAACATCGGGACTGCTCTGGCTAAGCTTTTTGTAGAGGCTCTTAGCTTTTCTGGACACCCTACCT
GTCTGGGTGTTGATGTGGCACTGTCCGTGAAGTGTCAGTATCTTGGAAAAGTTGTGCAGGATAT
GGCAGCGCTTATATTTGATAGCAGGCGGCGTATATGCTACACAGCATTGGTGAACCATTTTTAT
GTAAGTGGCGCCTTGAAGGAGCTCCTGACTACTTTTGAAGCCACTAGTCAGTTATTATGGTCCCT
ACCACACTCTATTCCAATGTCAGGTACTGACTCAGGGCAGGCAGGGAATGGAAGCAAAGTTGA
GCACAGTTCTTGGCTTCTTGATACTTTGCAGAGTTATTGCCGTCTGCTTGAATACTTTGTGAATTC
TTCTTTGCTTTTATCTACGAGTTCTCCAAATCAGGCTCAGCTGCTTGTTCAGCCAGTTGCCATTGG
GCTGTCAATTGGTTTGTTTCCTGTTCCAAGAGATCCAGAAGTCTTTGTGCGTATGCTCCAGTCTCA
AGTTCTTGACGTGGTACTTCCGGTGTGGAACCACCCCATGTTTCCTCACTGTAGTCCAGCCTTTA
TTGCTTCTGTTCTTTCCCTTGTAACTCACATATATTCTGGAGTTGGAGATGTGAAGCGCAGCCGT
AGTGGTGTGGCTGGAAGCTCAAACCAGCGTGTTGCTCCTCCCCTTGATGAAGCTGCAGTTGCCA
CCATTGTTGAGATGGGTTTTTCAAGGGCTAGAGCTGAGGAAGCCCTGAGACGAGTGGAGAGCA
ATAGTGTGGAGATGGCCATGGATTGGTTGTGCACTCATGCTGAAGATCCTGTGCAGGAGGATGA
CGAATTGGCGCGGGCTCTTGCATTATCACTTGGGGGTTCAAATGAAACTTCTAAGAGTGATGCT
ACGGACAAGTCAGTGGATATCCTGGGTGAAGAGGGGCAAATCAAGGTCCCTCCTGTTGATGAT
ATCCTTGGAGCATCAATGAAGTTGTTTCAGAGTGGAGATTCCTTAGCCTTTCCCTTGACTGATAT
GCTTGTGACACTGAGCAATCGCAACAAAGGTGAAGACCGTCCTAAAGTCACTTCTTATCTTGTT
CAGCGACTGAAACTTGGTTGCCCCGATTTGTCAAGAGATGCTGGTGCTGTGTCATCTATATCTCA
TATTTTGGCCTTGCTTCTTTCGGAAGATACTGGTGCTCGTGAAGTTGCTGCCCAGAGCGGTATTG
TTTCTGTTGCCATTGATATCTTGAAGAATTTTAGAATGAGCGACCAGCTTGGGAATGAGACATTC
ACAAAAAGCATAAGTGCTTTGCTACTCATATTGGACAATTTGCTGCAATCCAGACCGAGAACCT
CCTCTGAAAACTCAACAGTTGCAGGTCCACAAGATGATTTGTCTAAACAACATGCTTCTATTCT
GGTTTCAGAATCTGGCGTTGACAAAAGCTTGATCTCTGATGCCTGTGAAAAGGAGTTGGTCCTTT
CATTTGAGAAAATATTTGGAAAGTCTACAGGATATCTTTCTCTGGAGGAGAGTGGTTTAGTACT
GGAAATTGCTTGTGATATGCTGAAACAGCATGTTCATGCAATAATAATGCAGGCAGTGTTACAG
TTGTGTGCTCGGTTGACCAAAATACATACTTTTGCCTTGCAATTTCTTGAAAGTGGAGGCATGAG
TGCTTTGTTCAATCTTCCTAGTAAATGCTTCTTTTCTGGTTATGACACTCTGGCATCAGCAATTGT
TAGGCATTTGCTGGAGGATCCCCAAACTCTGCAAACTGCAATGGAGCTTGAGATTCGACAAACT
CTTAATGGAACCCGTCATGGTGGACGTATCCCCACCCGTGTGTTTTTGACTTCAATGGCACCTGT
TATCTCTCGAGATCCTGTGATATTTGCAAAAGCTGCTGCAGCAGTTTGTCAGTTGGAGTCATCTG
GAGGGAGGGCTGTTGTAGTTTTGTCCAAAGAAAAGGACAAGGACAGATCTAAATCAATTGGTG
CTGAGGGTGGATTATCATCTGGTGATTCCATTAGAATGCCTGAAATGAAGATCCAGGATGGGCC
AGGAAAGGGTTCCAAAGGCTGCAAAAAGATCCCCGCTAATTTGTCTCAAGTTATAGATCAGCTT
CTTGAAATAGTTCTGCAGTTCCCATCGTTGAATTTCCAGGAAGACTCTTTGAGCTTCTCAAGTGC
AATGGAAGTCGATCTACCTGCCACAAGTACGAAAGGTAAATCAAAAGTTGGTGAGGCAAATAA
AAAAGTCGAATCAGATTATCTACCTGAAAGATCAGCAGCTCTTGCTAAAGTGACGTTTGTTCTG
AAACTGTTAAGTGAGATTATTCTGATGTATGTGCATGCGGTTACGGTTGTATTAAAACGGGATTC

AGAACTTTGTCAGCTGCGAGGTTCTTGTCAGCTTGATACTTCTTCATCTGGGCCAGCAGGGGTAG
TACATCACGTTCTAAATAAGCTTCTTCCGGTATCAACGGATAGCTCTGTTGGACCTAGTGAGCTG
AGGGAAAAGTTGTCTGAGAAGGCGTCATGGTTTTTAGTTGTTTTGTGTGGGCGCTCAGGTGAAG
GGCGTAGACGAGTCATTAGTGAGCTTGTGAAGGCCTTGTCCTTGTACGGTGACTTGAAGAGCAA
TACTGGGAAAAGCAGTTTGCTACCAGATAAAAAGGTTCTGGGATTTGTGGAGTTGGTCTATTCT
GTTCTGTCAAAGAATTCCTCCTCCAATGGATTATCAGTTTCCGGTTATTCTCCTGACATTGCTCGA
AGCATGATAGATGGGGGAATTGTTCCATGTTTGACAAGCATTCTGCAGGAGATGGACTTGGACC
ATCCAGATGCTCCAAAAATTGCAAATATCATACTCAAGGCTTTGGAAAGCTTATCCCGTGCTGC
TAATGCGAGTGAACAAACCTGTAAATCTGATCCCCTGAACAAAAAAAGGTCTACGGGTGCAGA
AGGGAGATCTGGTGAGCAGACAGCATCTCCCAGTCAGACCACTGACCTTAATAATGACAATAG
GTCTGGTGAGCAGGAAGTCACCAATGCAGGGGTGAATGGGCAGAACCTCCCGGAAGAAGCTC
GTCATACTGAAAGCAATCATGGCTCTGGTGTGATCAGGTCAGCTGAGCAAGACATGAGGCATG
ATGGAGAAGGGCCTTCGATAGCGAACACACCGGGGGAGCATACAATGGAGTTCACACGTGAT
GGGATGGAAGATGCTGATCTAGTCCATAACGCAGGCCAAATTCATATGGATTTCCATGTTGAGA
ATCGAGCAGATGAAGATATCGGTGATGAGGATGATGATATGGGGGCTGAAGGTGAGGAGGAT
GATGATGATGAGGATGATGGCGAGGATGAGGATGAGGATATTGGCGGAGATGGAACTGCTCTT
ATGTCTCTTGCAGATACTGATGTGGACGATCATGAAGATACTGGGTTGGGAGATGAGTACAATG
ATGACATGATTGATGAAGACGAGTATCATGAGAACCGTGTCATAGAGGTAAGATGGAGGGAGG
CTTTGGATGGCTTTGATCATCTGCAGGTGGCTCAGTCTGCAAGTAGCTTTATTAATGCTGCTTCTG
AGCCTTTTGAGGGTGTAAATGTGGATGACCTCTTTAGTCTTAGGAGAACCTTGGGCTTTGAGAG
ACGGAGGCAAACAAGCAGGCCTTTCGAGCGAAGTAATACTGACGGAGGTATTCAACATCCTCT
GCTGTTGAGACAACCTCAATCTGGAAATGGTTCATTATGGTCATCAGGTGGCAATGCTTCCCGG
GATTCAGAGTCTATGATAGGTGGAGGCATTGATATATCACATTTCTATATGTTTGACTCTCCTGC
TCTCCCTTATGAATTTGGATCTGGCAGCTTGTTTGGTGATCGTTCAGATGGAACAGTGCCTCCGC
CGTTGACTGATTATTCAGTGGGTATGGATTCATTGCATATAGCTGGTCGTAGAGGGGCAGATGA
TGGTCGATGGGCTGATGATGGCCAGCCACAAGCCAGCAATCAAGCTGCTACTATAGCGCAGGC
TGTGGAGGAGGTGTTTGTTTCCCTTTTGCGCAGTGCTGCTTCTGCCAACACACCAGTTGGAAGAC
AGTCTCAGAATGCAGGGCAGCAGGAACAGCAATCAGATGCTCTGCAGAATAGCAATAATCATT
TGGAAGCTGGCGGAACTCATTCAGATGGACATAATAATGAGGGGCAGCTAGGAAATGGGAAT
GAAAATGACGTCCTACAAAGGGATGCATCAGATGTAGTTGCTGTTCAAGATCACGTCAATGCTA
CAGAGGAGGTTGCTAATGCTTCCCATGATGGTGAACCCTTATCAAGCAACCCCGAACAGTGTGA
CATGGATTCTGGACATGGAAATGGTCCATCAGGTGAACAAGCAGGTGCGATCCAAGAGCCATT
GCCATCATCTTCAGATCTTGAAAACAACCAGCATCTTGGAGGGGATTCTAGACTAATGGCTAAT
ATAGAAGATGAGCAAGTGAATGCTCCTGGAAACAATGAATTACAACCTTCGAATGATCTTGTA
GTTAATCCTGGAGAAGAGACAGTTTGTCCAATTACTGATCAGTCTTCTACTGTTCCTGCCAGCAC
TGATGTTGAGATGAATGGCACAGATGTTGAGCCTGCTCAACCTGATCATCCAACGCCCACGCCT
GAGGGTGATGAGCCTTCAGCTGATCATAATGTTTCAGTTAATCAACCCTCCATGGAGTCTGACC
GCAGCGGAGCTAATGGCGAACCACCTGGTCCCAGTGCAATTGATCCAACCTTCTTGGAGGCGCT
ACCTGAAGACTTGAGAGCTGAAGTGTTAGCTTCTCAACAATCTCAATCTGTGCAACCTCCTGCC
TATACACCACCATCTGTAGAAGACATTGATCCTGAGTTTCTGGCTGCACTACCTCCTGATATTCA
AGCAGAAGTACTGGCCCAGCAACGAGCCCAGAGATTGGCTCAGCAGAGTGAAGGACAACCAG
TTGACATGGATAATGCTTCTATAATTGCTACACTTCCTGCTGATTTGCGTGAGGAGGTTCTTTTGA
CGTCTTCTGAAGCCGTTCTATCAGCTTTGCCTTCTCCATTGCTTGCTGAGGCGCAAATGCTTAGG
GATAGAGCAATGAGCCACTACCAGGCTCGCAGCCTTTTTGGAAGCAGTCACAGATTGACTAAT

CGAAGAAATGGTTTGGGCTTTGATAGGCAGTCAGTTATGGATCGGGGTGTTGGAGTAACCATAG
GACGAAGGACGGGGTCTTCGCTCTCAGAGGTCTTGAAAGTGAAAGAAGTTGAAGGCGAGCCAC
TCCTTGATGCCAATGCACTGAAGGCATTAATCAGGCTTTTACGGTTGGCACAGCCTCTTGGTAA
AGGCCTTCTGCAGAGACTTTTGTTGAACTTGTGTGCTCACAGTGTCACTAGAGCAAATCTTGTTC
ATCTTTTGCTGGGTATGATCAAGCCTGAAGCTGAAGGCACTGCCGGAAATCTCTCAGTGGTCAA
TCCTCAGAGGCTTTATGGCTGTCCACCAAATATTGTGTATGGTCAATCCCAGTTGTTAGATGGTC
TCCCACCTTTGGTTTTGCGACGTGTTCTTGAGGTTTTGACTTATTTGGCCACAAATCATTCTGCTG
TTGCAAATACCTTGTTCTATTTTGAACCTTCACTTCTACCTGAGTCCTCTGAAAACTTCTTGGAGA
CAAAGAATGGTAAGGGCAAGGAGAAAGTTGACGAAGGTGGAATGAGTTCAAACTCGCATGGA
GCTCTGCGTGAAGGAAGTATTCCTGTGATATTGTTACTAAAGCTTCTAAAGTGCCCCCTTTTCCG
GCGCAGCATTGCTCATGTTGAGCAGGTTATGGGTCTGCTTCAAGTGGTCATATACACTGCAGCTT
CAAGATTAGAAAGTCTTTCCCCCCCGGATAAGGTCTCAGATGATGCCAAGAATTTGAGTGTTAA
AGCCTCTGATGGACCACCAGGAGAAGATGCTTTAGAAACAGAGACTAAGCAAAAAGGTCCTGT
GGAGAAGGGAGAATCATCTTCATCTGACAGTCCAAGAGGCCATAATATTCATGATGTTTTTGTT
CAGCTTCCCAAACCTGACCTGCGAAATCTCTGTCGGCTTCTTGGCTATGAAGGGCTGTCAGATA
AAGTGTATATGCTTGCTGGAGAAGTGCTGAAAAAGTTGGCCACCGTCGTCGCTGCCCACCGCAA
ATTCTTCACTCTAGAGCTTTCTGATCTTGCTCAAGACTTGAGCAGTGCTGCAGTGGCTGAGCTAA
CTACCCTTAAAAACACATGCATGATGGGTCTTAGTGCTGGTTCTATGGCTGGTGCTGCCATGCTG
CGTGTACTGCAAGCTCTTAGTTCCCTCATTTCGCACAATTTTGATGACAGTTGCAGTCAGGAGAA
TGGCGAGGAGCATGAGGAGCAAGTTGTAATGCTGAAGTTGAATTCTGCTCTGGAGCCATTGTGG
CAGGAACTCAGTGAATGCATAACTGCAGCTGAGTCAGAATTGGGACAGGCCCCCTTATCGCCA
ATCATAGCTACTATGGGTGCTGGGGATAATGTTGCAGGGTCCTCTCCTTCATCTCCTCCTCTCCCT
CCTGGGACCCAAAGATTGCTGCCCTTTATCGAGGCTTTCTTTGTTCTTTGTGAGAAGCTGCAATC
GAGCAATTCACTGCTGCAGCAGGATCAATCCAATGTTACTGCACGGGAAGTTAAAGAATCTGCT
AGTAGTTGTCCATCAGCGCTGTCTACGAGTGGTGGAGATTCTCAGAGAAGATTTGATAGTGCAA
CCATATTTTCAAGGTTTGCCGAAAAACATCGGCGGCTTTTGAATGCATTTATTAGGCAGAATCC
AGGGTTGCTTGAGAAGTCACTCTCTTTGATGCTAAAGGCTCCTCGCCTCATCGACTTTGATAACA
AGAGGGCATATTTCCGCTCCAGAATCCGACAACAACATGAGCAACACCTTGCAGGGCCTCTCA
GGATTAGTGTTCGTCGGGCATATGTTCTTGAAGACTCATATAATCAACTGCGTATGAGGTCTACT
CAGGACCTGAAAGGGCGGTTGAATGTGCAGTTCCAAGGTGAGGAGGGTATTGATGCTGGTGGC
CTCACAAGAGAGTGGTATCAACTTTTGTCAAGAGTCATCTTTGATAAGGGAGCTCTGCTATTCA
CCACTGTTGGAAGCAATGCAACTTTCCAACCAAACCCGAATTCTGTATACCAGACTGAACATCT
GTCATACTTCAAGTTTGTTGGCCGTGTGGTTGCCAAAGCGCTATTTGACGGGCAACTTTTGGATG
TGTACTTCACTCGTTCTTTTTACAAGCATATTCTGGGTGTGAAGGTGACCTACCATGATATTGAG
GCTGTTGATCCTGATTACTATAAAAACTTGAAGTGGATGCTCGAGAATGATGTCAGTGATATTCT
TGACCTAACTTTTAGCATGGATGCGGATGAGGAAAAGCACATTTTGTATGGGAAAACAGAGGT
TACTGATTATGAGCTCAAGCCTGGAGGAAGAAACATCAGGGTTACAGAGGAAACAAAGCATG
AGTATGTAGATCTTGTTGCTGATCATATTTTGACAAATGCCATCCGTCCCCAGATCAATTCGTTC
CTTGAAGGTTTTAATGAGCTAGTGCCTCGAGAGCTGATTTCTATTTTCAATGACAAGGAGCTTGA
GCTCCTTATAAGTGGACTTCCCGAGATCGACTTGGATGATTTGAAGGCGAACACCGAGTATACT
GGCTACACAGCCGCATCTAATGTCATTCAGTGGTTTTGGGAGGTTGTTAATAGTTTCACTAAAGA
AGATATGGCCAGATTTCTGCAATTTGTCACTGGCACTTCTAAGGTCCCATTAGAGGGGTTCAAA
GCATTGCAAGGCATCTCAGGTCCCCAAAGATTTCAGATTCACAAGGCATATGGAACTCCAGAG

CGACTGCCTTCGGCTCATACATGTTTCAACCAATTGGACCTCCCTGAGTATGCTTCGAAAGAGC
AGCTTAAAGAGCGTCTGTTGCTCGCTATTCATGAAGCTAGCGAAGGCTTTGGCTTTGGTTGA

>PolA05G021080.1 gene name=Ubi-2
ATGGACAATAAAGAGGAAGTTGCTGTGGATGCTACACCCAGCAAGACTGCGGAAGGGACCTCT
GTGTTCCGTGAAATTGAGTTCCATTTGGCTAAAAAATCACCTTCTTTTTCTAAAGCCTCTGATTTC
AAGCTTGAAACTTTGAACCCATCCACGTCCTCTAAATCTCCTCTATCCCACTCCCCCTCTTCATCT
AGAAAGGCAGAGGTCTCTGATTTTCTTGATAGCCTGCTTGATCCTGAGCTCACTTTCAGAATCAC
TTACCGAAGGATTGGTGCTGGTTTGGAAAATCTTGGGAATACATGCTTCCTTAACTCTGTGCTTC
AGTGCCTGACATACACAGAGCCATTAGTAGCATATCTGCAAAGTGGCAAGCACCAAACTAATT
GTCACATAGCTGGTTTCTGTGCTCTATGCGCCATCCAGAGACATGTCAGCCGTGCACTGCAGTCT
ACAGGGAGAATAGTGGCACCAAAAGACCTCGTCTCAAATTTGAGATGCATATCCCGGAACTTT
CGAAATGCAAGACAAGAGGATGCACATGAATACATGATACATCTGTTGGAATCAATGCACAAA
TGCTGCTTACCCTCAGGGGTACCTGCCCAATCACCTGGTGCTTATGAAAAAAGTTTGGTTCACA
AGATCTTTGGTGGCCGCCTTCGCAGTCAGGTGAAATGCTTGCAATGTAACTATTGTTCGAACAC
ATTTGATCCTTTCTTGGATCTTAGCCTTGAAATAGCAAAGGCTGATTCTCTATATAAGGCCCTTG
CTCATTTCACTGCTGCTGAACAACTGGACGGCGGAGAAAGGCAGTACAAGTGTGAAAGGTGCA
AGCATAAAGTTCGGGCACTCAAACAGCTCACTATTTACCAAGCACCATACATTCTTTCTGTCCA
TCTAAAGCGTTTTCGTTCTCACTTTGGTGGGCAAAAGATTGACAAGAAAGTTCAGTTTGGTTCTA
CATTGGATCTGAAGCCTTTCGTCACTGGACCCTATGAGGGTGATCTCAAATACACACTGTACGG
AGTTCTGGTTCATGCTGGTTGGAGCACACATTCTGGTCACTACTATTGCTTTGTCCGCACTTCAA
GTGGATTATGGTACAAGCTTGATGATAATACGGTCGCTCCAGTGAGTGAGAAGTCTGTCCTGGA
GCAGAAAGCATACATGTTATTTTATGTTCGTGACAGGAAGAACTTTGTTCCCAAGAAAGCCTTA
GATATTGTTCACAAGGAAAGCTTGATCACAACTATGCGCAAGGCTTCGGGATCTTGCTCTGTGC
CCAATGTTAAGGAGTTTCAAAGTGATCGTACTCCTGGGAATCCAAGTGAAAAAGTTCCATCCGG
TTCTGATATGAAGACCTCCGGCAACATTGACACTTTTGTGAAGGATTTGAATCAGAAAAAGGCT
GATGATACAAAGTCTACCGCACCCACAGCTTTGAAAAGCTCTAAAGCCACAGGTGATGTGGTT
GGTGCTAGCTGCAATGGAAAGGTCCAGGATCAAGCGTCCGCCACCAAACCTTCAAGTCTTCAC
GATTCATCTACTGCTTCTGGTGATGGGGGCAACAAAAATTCTCATGTGCTAAATACTCAACCAC
CAAATGTTGCTGCGGACACGAAGGACGTACCTTGTCGTGAGAATGATTGTAAGTTGGTGAAAA
ATGACCCACCTCTGTCAGCAACTCAACAGAACACTGGAAGTGAAATCTGCAAGGAGGAAACTG
TTGCGCCCAAGGAAGAGATGCAGCCAACCGATGCCAAATCAAGACGTAAGATGTCAATTATGA
ATGGATCTTCCAAGCGATCCGATCGCACTCAACGTGGAAAATTAATGAAGAAGATTCTGAAGA
GTAGAGTTGGGACGCATCTTAGTACCAAGATGTTGTATGGAGCATCTCTAAGCTTAAAGAAGAG
GAGATGCAAAACCAGAAAACTGAACCATAAAAGTGTTCTACACGAAGACTCGGTTGCTAAAGC
TATGGTCTCTGTAGATGTGGTGGGTCCTGCAACATTTGAGAAAAAGGTGGAAGCTTCAGTGATT
GATGAGGCTCCACAAGCACGTGGGAGCTCCAAAGCAGATCACGTGGGTGTTGGTGCCTCTAGTT
CTTCTCAACCACAAAATGGAGTAACTCCTGCTGCCACCGTTGTATTCAAGGAGACTACTGTTAC
ACGTTGGGAGGGAGTAGACACACCTTCAGAGACTGCACGATCAAGTGGTCCAAAAACGTCGAG
CATTGGTTATGTAGGAGATGAGTGGGATGAGGAATACGACCGTGGTAAAAGGAAGAAGGTAA
GAGGGCCGAGACACGATTTCAGCGGACCAAACCTCTTCCAAGAAGTTGCATCACAGAAAGCAA
AACGAAGGCGACAAAACTAA

>PolA08G004380.1 gene name=eIF5A

ATGTCGGACGAAGAGCATCACTTTGAGTCCAAGGCCGATGCTGGTGCATCTAAGACCTACCCTC
AACAGGCTGGAACCATCCGTAAGAATGGTTACATCGTCATCAAGAATCGCCCTTGCAAGGTTGT
TGAGGTTTCCACTTCAAAGACTGGGAAGCACGGTCACGCTAAGTGTCACTTTGTAGGAATCGAC
ATTTTCACCGGAAAGAAGCTCGAGGATATTGTTCCATCATCCCACAACTGTGATGTTCCTCATGT
CAACCGTACTGATTATCAGCTGATTGATATCTCTGAAGATGGTTTTGTTAGCCTTCTTACTGAGA
ATGGAAATACTAAGGATGATTTGAGACTTCCTACTGATGATGCATTGCTTGCTCAGATTAAGAG
TGGCTTTGAGGAAGGGAAGGACCTCATTGTTAGCGTCCAGTCGGCCATGGGAGAAGAGCAGAT
TTGCGGTCTCAAGGACATTGGAAAGAATTAG

Figure S2. CDS sequences of candidate reference genes. Coding DNA sequences (CDS) of the candidate reference genes in *Portulaca oleracea* and the sequence identifiers (gene names and gene IDs) correspond to Table 1.
